# Total serum *N*-glycans mark visceral leishmaniasis in human infections with *Leishmania infantum*

**DOI:** 10.1016/j.isci.2023.107021

**Published:** 2023-06-05

**Authors:** Gabriane Nascimento Porcino, Marco René Bladergroen, Viktoria Dotz, Simone Nicolardi, Elham Memarian, Luiz Gustavo Gardinassi, Carlos Henrique Nery Costa, Roque Pacheco de Almeida, Isabel Kinney Ferreira de Miranda Santos, Manfred Wuhrer

**Affiliations:** 1 Departamento de Bioquímica e Imunologia, Faculdade de Medicina de Ribeirão Preto, Universidade de São Paulo, Ribeirão Preto 14049-900, Brazil; 2 Center for Proteomics and Metabolomics, Leiden University Medical Center, Leiden 2333 ZA, the Netherlands; 3 Instituto de Patologia Tropical e Saúde Pública, Universidade Federal de Goiás, Goiânia 74605-050, Brazil; 4 Instituto de Doenças Tropicais Natan Portela, Universidade Federal do Piauí, Teresina 64002-510, Brazil; 5 Departamento de Medicina, Programa de Pós-Graduação em Ciências da Saúde – PPGCS, Universidade Federal de Sergipe, Aracajú 49060-100, Brazil

**Keywords:** Glycobiology, Pathophysiology, Glycomics

## Abstract

Visceral leishmaniasis (VL) is a clinical form of leishmaniasis with high mortality rates when not treated. Diagnosis suffers from invasive techniques and sub-optimal sensitivities. The current (affordable) treatment with pentavalent antimony as advised by the WHO is possibly harmful to the patient. There is need for an improved diagnosis to prevent possibly unnecessary treatment. *N*-glycan analysis may aid in diagnosis. We evaluated the *N*-glycan profiles from active VL, asymptomatic infections (ASYMP) and controls from non-endemic (NC) and endemic (EC) areas. Active VL has a distinct *N*-glycome profile that associates with disease severity. Our study suggests that the observed glycan signatures could be a valuable additive to diagnosis and assist in identifying possible markers of disease and understanding the pathogenesis of VL. Further studies are warranted to assess a possible future role of blood glycome analysis in active VL diagnosis and should aim at disease specificity.

## Highlights

• Visceral leishmaniasis (VL) has a distinct *N*-glycome profile
• The *N*-glycome profile of VL reflects treatment response
• The *N*-glycome of asymptomatic infections doesn’t differ from that of healthy controls
• *N*-glycome analysis has potential as an alternative to invasive diagnostic techniques

## Graphical abstract

**Figure:**
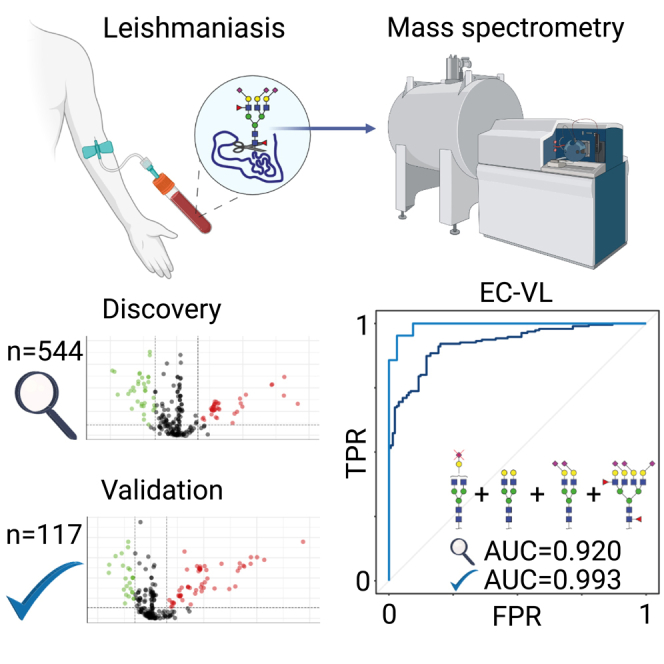

## Introduction

Leishmaniasis is a major neglected tropical disease and a global public health problem since it is present in 98 countries of the world, with 700,000 to one million new cases every year and an estimated 350 million people at risk of infection (^1^^,^^2^ and https://www.who.int/news-room/fact-sheets/detail/leishmaniasis, accessed on Dec 2, 2022). Within the Americas the disease is present in 19 countries of which Brazil is the most affected country. The three main clinical forms are cutaneous (CL), mucosal (ML) and visceral leishmaniasis (VL). VL presents high mortality rates in patients being fatal in 95% of cases when not treated^3^ and is the clinical form this study is focused on.

*Leishmania infantum* is the causative agent of VL in Brazil.^4^ The disease was first recognized in the country in 1932^5^ probably taken to the northeast region of Brazil by people or dogs from southern Europe or North Africa infected with the parasite.^6^ The competent vector for *L. infantum* found in most countries in Latin America is the sandfly species *Lutzomyia longipalpis* and major reservoirs of *L. infantum* are dogs.^7^^,^^8^ In addition, a role of asymptomatically infected humans as reservoirs for *L. infantum* has been suggested.^9^^,^^10^

VL can be classified as an opportunistic infection because it is a co-infection in many HIV patients^11^ and it is known that HIV infection massively amplifies the susceptibility as well as the severity of VL.^12^ The susceptibility factors for VL include age – with children under one year old and adults above 50 years being the most affected^13^ – as well as genetic background of the host,^14^ nutritional status,^15^ sex,^16^ and immune suppression.^17^

For asymptomatic infected individuals, it is difficult to predict whether and when the disease will become active, with environmental, parasitic and host-related factors playing a role.^18^ The asymptomatic incubation period of VL has a variable duration and intermittent fever, malaise and shivering are included as early symptoms. Splenomegaly, accompanied or not by hepatomegaly, are symptoms manifested in overt disease.^19^^,^^20^

The gold standard for diagnosis of VL is the parasitological exam with visualization of the amastigote, a motile, round and obligate form of the *L. infantum,* in biopsied material which should preferably be obtained from aspirates or biopsies of bone marrow, lymph nodes or spleen. The procedures must be performed in a hospital environment under surgical conditions, then the sample can be purposed for smears and examined after Giemsa staining or analyzed by molecular biology tests.^21^^,^^22^ Disadvantages of the bone marrow puncture used in Europe, Brazil, and the US are the difficulty to perform the procedure, the inflicted pain and the risk of fatal bleeding, although this method has lower risks compared to spleen aspirates primarily performed in eastern Africa and on the Indian subcontinent. This perhaps influences the choice in favor of the bone marrow aspiration by some countries, despite the lower sensitivity (60–85%).^23^^,^^24^ Given the invasive nature of VL diagnosis and the associated risks of morbidity and even mortality, there is a need for alternative or complementary, preferably non-invasive diagnostic approaches.

When analyzing samples obtained in a non-invasive manner (peripheral blood, buffy coat, and peripheral blood mononuclear cells (PBMCs)) microscopy has lower sensitivity than culture, whereas the complete diagnostic result with culture can take from a few days to weeks.^25^ The low invasive rK39 rapid strip immunochromatographic test exhibits varying sensitivities and specificities in different geographic regions compromising its usefulness for diagnosis.^24^

There is no standard diagnosis to detect asymptomatic infections with high sensitivity. One or more different and possibly complementary techniques are needed to detect the infection,^26^ but these are still moderate in sensitivity. More research is needed to identify ideal biomarkers for both symptomatic and asymptomatic VL to detect the disease in an early stage with high sensitivity and minimal invasion. One of these markers might be profiles of *N*-glycans on serum proteins, including Immunoglobulin G (IgG) and other antibodies.

*N*-glycans are sugar chains covalently linked to asparagine residues on proteins.^27^*N*-glycan profiles profoundly affect the biological functions of protein secretion, trafficking, receptor interaction and modulation of the immune response^28^ and can serve as biomarkers for early diagnosis and patient stratification.^29^ Several common features within these glycans, known as glycosylation traits, were shown to be related to specific biological functions. As an example, we would like to mention that lower fucosylation, lower galactosylation, and higher sialylation in combination with an increase in large-size glycans and a decrease of hybrid and high-mannose structures were correlated with inflammatory bowel disease (IBD).^30^

Over the years, improvements have been made in the techniques for glycan analysis which nowadays provide valuable tools in research and clinical biomarker development.^31^

Although glycan signatures are highly stable within an individual reflecting its physiological stage,^32^ many diseases such as cancer and autoimmune disease affect glycosylation.^33^^,^^34^ Similarly, with aging, hormonal changes and pregnancy glycosylation is changing.^35^^,^^36^^,^^37^

A previous study has shown that VL patients produce IgG with Fc glycosylation patterns like those found in other inflammatory conditions. Accordingly, IgG Fc *N*-glycosylation associates with serum cytokines levels, C-reactive protein as well as disease severity.^38^ The glycosylation of other serum proteins was, however, not studied.

Here, we analyzed total serum *N*-glycosylation in Visceral leishmaniasis using a high-throughput technique.^39^^,^^40^^,^^41^ We explored associations of total serum *N*-glycosylation and VL with regards to presence or absence of symptoms, severity of disease and post-treatment recovery to obtain insights into potential new markers for diagnosis and therapy monitoring.

## Results

### The analytical pathway

**Figure 1:**
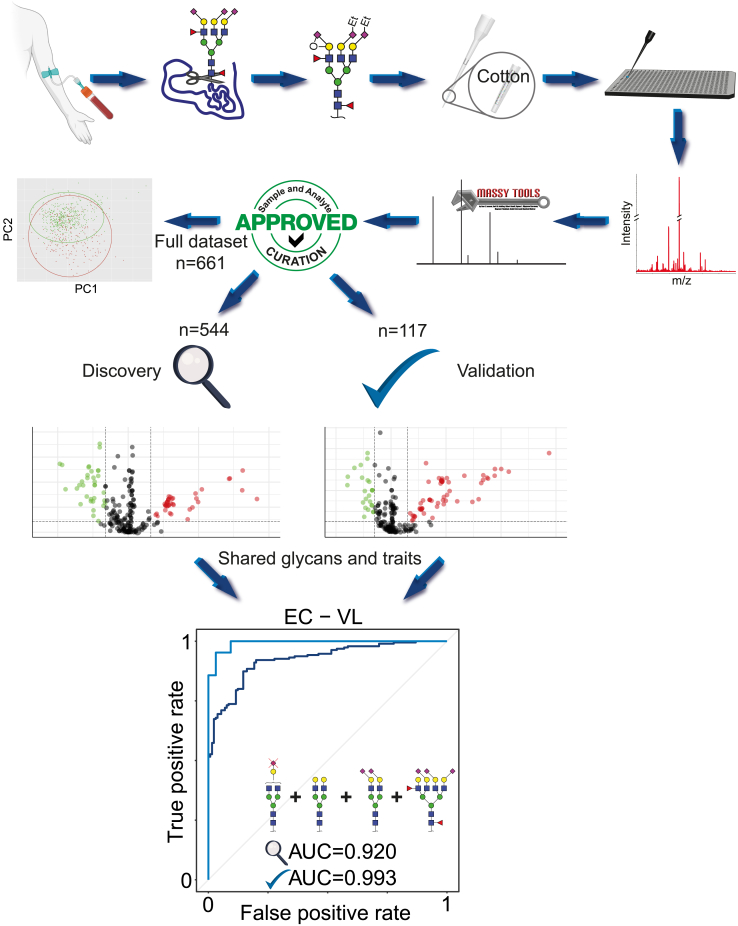
Graphical flow-chart of the analytical methods used in this research After collection of serum (or plasma) from the participants in this study the glycans were released from the proteins, derivatized on the sialic acids to accommodate glycan isomer differentiation, purified using cotton HILIC and measured with a mass spectrometer. The data were processed using MassyTools software and data curation was performed to eliminate low quality data. A PCA analysis was performed on the full dataset, after which the data was split into a discovery and a validation set as described in the STAR Methods section. After analyte selection using a Wilcoxon rank-sum test, a model was built that was able to distinguish Visceral Leishmaniasis (VL) patients from Healthy endemic control individuals (EC). Data are represented both as box plots, which show the 0th and 100th percentiles, the sample median, and the first and third quartiles, and dots representing values for individuals.

A graphical representation of the analytical pathway followed is depicted in Figure 1. The serum and/or plasma *N*-glycomes from patients diagnosed with VL together with samples from control individuals were analyzed by mass spectrometry. Of the initial 756 collected samples, after spectra and analyte curation, a total of 661 serum samples characterized by 73 glycans were included in the study. Exclusions were based on parameters as described in the STAR Methods section. As described in this same section, a large subset of these samples (n = 544) was collected between 2012 and 2013 and used as the discovery set. The remaining samples were collected in 2018 and used as a validation set. Also, 76 spectra from plasma at various timepoints after treatment were included in a longitudinal study. The characteristics of the participants cohort are summarized in Table 1.

**Table 1:** Cohort information

Group^a^	Discovery (2012–2013)^b^				
	Total no.	Mean age, yr. (SD)	No. (%) of:		
			Females	Males	NR^f^
VL	190	25.7 (17.8)	68 (36)	111 (58)	11 (6)
ASYMP^g^	177	36.5 (17.8)	104 (59)	58 (33)	15 (8)
EC^h^	130	36.0 (18.3)	80 (62)	29 (22)	21 (16)
NC^i^	47	30.6 (10.9)	19 (40)	16 (34)	12 (26)

	Validation (2018)^b^				
	Total no.	Mean age, yr. (SD)	No. (%) of:		
			Females	Males	NR^f^
VL	21	42.7 (15.7)	2 (10)	19 (90)	0 (0)
ASYMP^g^	52	39.3 (14.0)	39 (75)	13 (25)	0 (0)
EC^h^	32	34.6 (10.6)	20 (63)	12 (37)	0 (0)
NC^i^	12	32.3 (10.5)	6 (50)	6 (50)	0 (0)

Day^d^	Treated VL patient plasma^c^				
	Total no.	Mean age, yr. (SD)	No. (%) of:		
			Females	Males	NR^f^
0	23	33.7 (12.6)	10 (44)	9 (39)	4(17)
5	22	33.7 (13.0)	9 (41)	9 (41)	4 (18)
90	19	35.3 (13.5)	7 (37)	8 (42)	4 (21)
180	12	33.5 (11.0)	6 (50)	4 (33)	2 (17)

	Clinical severity^e^				
	Total no.	Mean age, yr. (SD)	No. (%) of:		
			Females	Males	NR^f^
U-VL	49	25.8 (17.9)	68 (36)	110 (58)	11 (6)
C-VL	82	29.1 (9.1)	17 (38)	16 (35)	12 (27)

a Serum sample-groups: healthy individuals from a non-endemic area (NC) living in Ribeirão Preto – São Paulo, southeast of Brazil, healthy individuals from an endemic area (EC), asymptomatic (ASYMP) individuals, and active Visceral Leishmaniasis patients (VL) living in Teresina – Piauí or Aracajú – Sergipe, regions from the northeast of Brazil.

b The discovery cohort was collected in 2012–2013. The VL samples from this cohort were collected from both regions as indicated above. The validation cohort was collected in 2018, with VL samples from Teresina only.

c Plasma samples for a longitudinal study from Visceral Leishmaniasis patients (VL) living in Teresina – Piauí or Aracajú – Sergipe, regions from the northeast of Brazil and collected during the same period as the discovery cohort.

d The table is showing the number of days (0, 5, 90, 180) after the beginning of treatment.

e The clinical severity of Visceral Leishmaniasis is separated into categories: U-VL uncomplicated; C-VL with complications that require the use of additional therapy to treat the patient, which include antibiotics or blood products, with hemorrhage and increased risk of death, according to laboratory data.

f NR means individuals whose sex or age has not been registered.

g The asymptomatic individuals were identified by positive tests according to literature.^26^^,^^38^ The same methods were applied to identify controls as.

h Healthy individuals from an endemic area.

i Healthy individuals from a non-endemic area through negative result.

For repeatability testing, 27 pooled serum samples and 67 VisuCon plasma samples spread over ten sample plates were analyzed regarding the 25 most abundant glycans, revealing an average overall CV of 17.1% (Figure S1A).

To support the validity of the data and because the sex distribution of our samples was unbalanced between the groups, trends of glycans known to be influenced by sex^39^^,^^42^ were evaluated and proven to be in accordance with the expectations (Figure S1B).

### The total serum *N*-glycome can distinguish VL from individuals without symptoms

Principal Component Analysis (PCA) was performed to explore the Total Serum *N*-Glycome (TSNG) of asymptomatic, VL and control groups. The summary of fit is shown in Figure S2A, showing that the first five principal components already describe a large portion of the variance in the data. The PCA showed that TSNG can distinguish VL from the other three groups of interest. Asymptomatic patients largely overlapped with healthy individuals. The slightly disbalanced sex distribution was found to be no major confounder of the disease signature. Also age, another factor that is known to be of influence in the expression of certain glycans,^43^ did not demonstrate any trends that would indicate confounding (Figure S2) as age was distributed evenly over the biological groups.

**Figure 2:**
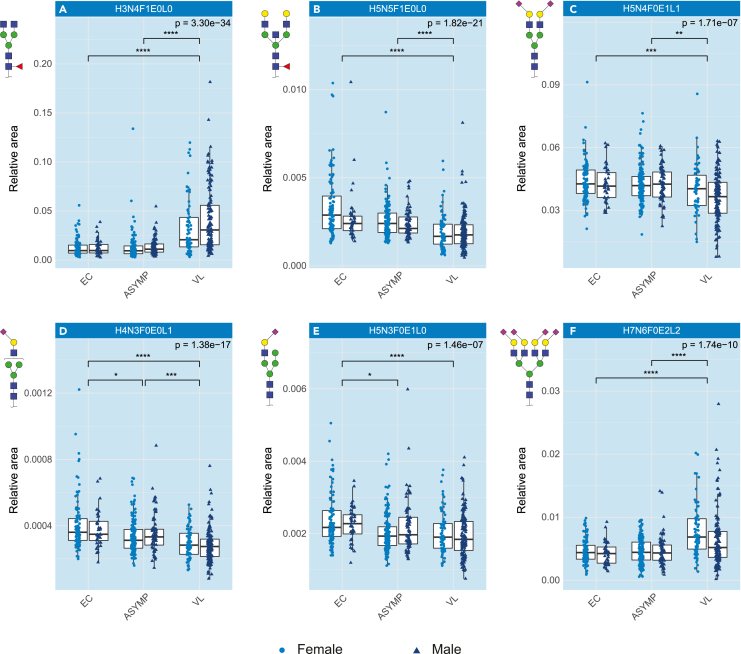
*N*-Glycosylation patterns are altered in VL Boxplots of a representative set of changed glycans (relative abundance) in the *N*-glycome signature in active visceral leishmaniasis, separated by sex. The error bars indicate variability outside the first and third quantiles around the median (bold midline) of the full dataset. The individual samples in the plots are represented by dark blue triangles for males and light blue circles for females. (A-F) Fucosylation is increasing in VL patients; (B) Bisection is decreasing in VL patients; (C) Sialylation, both (D) 2,3-linked and (E) 2,6-linked, are decreasing in VL patients, however, not on the tetra-antennary glycans; (F) Tetra-antennary glycans, which are highly sialylated, are increasing in VL patients. The analysis was performed on a total of 73 glycans using a Kruskal-Wallis test and post-hoc Dunn’s test with a significance threshold of α = 5.0e-5. Significant differences are indicated with ∗p < 5.0e-5, ∗∗p < 1.0e-5, ∗∗∗p < 1.0e-6, ∗∗∗∗p < 1.0e-7. EC = Endemic Control, ASYMP = Asymptomatic and VL = Visceral Leishmaniasis. H = hexose, N=N-acetylhexosamine, F = deoxyhexose (fucose), L = lactonized N-acetylneuraminic acid (α2,3-linked), E = ethyl esterified N-acetylneuraminic acid (α2,6-linked). See also Figure S3.

Sialylation of diantennary structures determines the first dimension of the PCA (Figure S2B), but its influence on disease remains unclear, since most of the discrimination in the PCA score plot is visible in the second dimension. Tri- and tetra-antennary structures drive the separation in the second dimension with fucosylated glycans indicating VL patients, whereas afucosylated glycans marked the non-manifesting individuals (NC, EC, and ASYMP). To investigate the differences between the VL patients and the other groups in more detail, the glycans were individually tested with a Kruskal-Wallis test (Figures 2, S3, and Table S3A) showing consistent signatures for males and females.

### Few glycan differences were observed in onset of symptoms

To explore the differences in the four groups of interest in more detail by means of univariate analysis, the sample cohort was split into a discovery and a validation set (see STAR Methods).

To obtain more insights into the possible onset of symptoms we first focused on the two healthy control groups using a Wilcoxon rank-sum test. No consistent, replicated differences were found between the endemic and non-endemic healthy control groups.

Three glycans were different between non-endemic controls and asymptomatic for both discovery and validation set. They all belong to the diantennary, bisected glycans. Of interest, no replicated glycosylation differences were observed when comparing endemic controls with asymptomatic persons. The observed differences in glycans are reflected in the significantly different glycosylation traits of hybrid, mono- and diantennary glycans (Tables S3C and S3D).

### Distinct *N*-glycome signature changes in active visceral leishmaniasis disease

**Figure 3:**
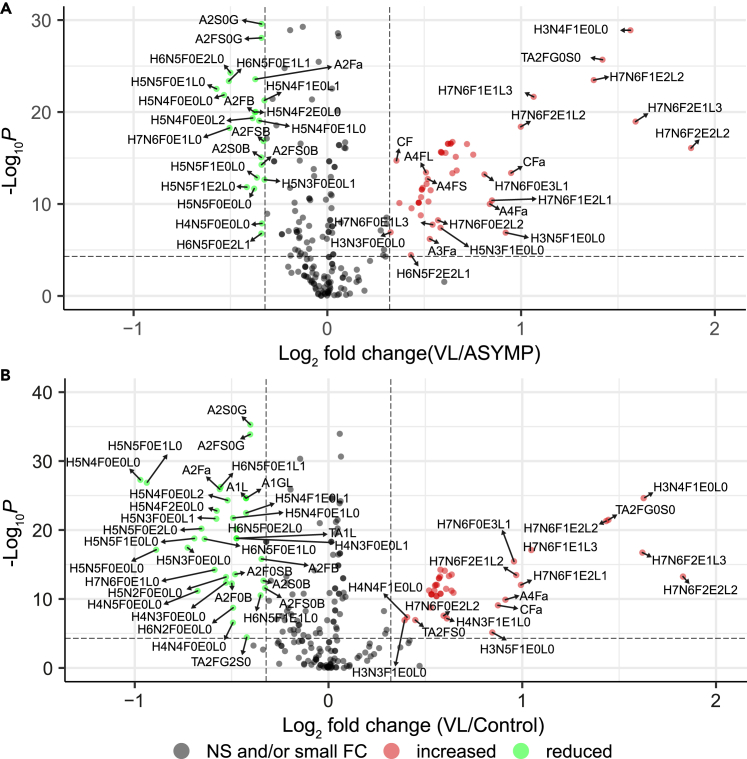
Differentially expressed glycosylation traits in VL (A and B) Differential glycan expression between (A) Asymptomatic (ASYMP) and Visceral Leishmaniasis (VL) and (B) Endemic Control and VL on the total dataset. Values on the x- and y axes show log_2_(fold change) vs. -log_10_(p value) of a Wilcoxon rank-sum test respectively. Green dots indicate significant decrease (α = 5.0e-5) with at least an absolute log_2_(fold-change) of 0.3, while red dots indicate significant increase with sufficient fold-change. Gray dots indicate no significant or sufficient change. The analysis was performed on a total of 226 variables (glycans and glycosylation traits). The complete table of the Wilcoxon tests can be found in Table S3. H = hexose, N=N-acetylhexosamine, F = deoxyhexose (fucose), L = lactonized N-acetylneuraminic acid (α2,3-linked), E = ethyl esterified N-acetylneuraminic acid (α2,6-linked), G = galactose, S = sialic acid, A = antenna, C = complex, T = total. See also Figure S4.

The majority of differences in this dataset are observed between VL-patients and the other groups (without clinical manifestations) (Figure 3; Tables S3E and S3F). Because the differences between non-endemic and endemic controls are not disease-related and the endemic controls are more closely related to the asymptomatic cases and to active VL compared to the non-endemic controls this latter group was disregarded in subsequent analyses. The Wilcoxon tests revealed 44 replicated glycans and glycosylation traits as significantly and sufficiently different between either EC or ASYMP and VL, in both the discovery and validation datasets (Table 2). Fucosylation was increased in samples from VL patients (represented by e.g., Figures 2A and S3 trait CF) compared to the other groups, mainly driven by core fucosylation, although antenna fucosylation also slightly increased on tri- and tetra-antennary glycans (Figure S3 traits A3F, A3Fa, A4F and A4Fa). Examination of the results in more detail showed that bisection of diantennary structures was reduced (Figures 2B and S3 trait CB), even in the presence of fucosylation. Overall sialylation decreased in VL (Figures 2C and S3 trait CS) with α2,6-linked sialylation as the main contributor to this decrease (Figure S3 trait CE), although α2,3-linked sialylation also was reduced, especially on the mono- and diantennary glycans (Figures 2D, 2E, and S3 traits TA2E, TA1L and TA2L). Opposing this trend, sialylation on the tetra-antennary glycans increased in VL patients (Figures 2F and S3 trait A4S), concurrent with an increase in tetra-antennary glycans in general (Figure S3 trait TA4).

**Table 2:** Wilcoxon test results on the 44 shared glycans and glycosylation traits

Endemic Control (n=162) versus Visceral Leishmaniasis (n=211)							Asymptomatic (n=229) versus Visceral Leishmaniasis (n=211)					
trait	estimate	statistic	p value	95% confidence interval	fold change VL/EC	Abs(-Log_2_(FC))	estimate	statistic	p value	95% confidence interval	fold change VL/ASYMP	Abs(-Log_2_(FC))
H7N6F2E2L2	−0.000333959	9332	5.61268E-14	−4.234E-04, -2.028E-04	3.554172646	1.829513763	−0.000324287	13062	8.25232E-17	−4.276E-04, -2.361E-04	3.673516604	1.877161796
H3N4F1E0L0	−0.015903486	6351	2.35371E-25	−2.090E-02, -1.178E-02	3.087935911	1.62664281	−0.015679955	9098	1.27851E-29	−2.045E-02, -1.193E-02	2.957398989	1.564328894
H7N6F2E1L3	−0.000472738	8316	1.87697E-17	−6.031E-04, -3.851E-04	3.073914738	1.620077149	−0.000469817	12060	1.09077E-19	−5.862E-04, -3.449E-04	3.012191827	1.590813649
TA2FG0S0	−0.017082789	7090	3.35885E-22	−2.297E-02, -1.278E-02	2.72252068	1.444943007	−0.017116755	9986	2.02866E-26	−2.243E-02, -1.320E-02	2.676110612	1.420137748
H7N6F1E2L2	−0.001436856	7132	4.99858E-22	−1.802E-03, -1.126E-03	2.700436606	1.433192681	−0.001372454	10635	3.35236E-24	−1.706E-03, -1.093E-03	2.593353639	1.374818951
H7N6F1E1L3	−0.001426651	8214	7.97244E-18	−1.774E-03, -1.052E-03	2.066125802	1.046928099	−0.001450392	11190	2.19409E-22	−1.771E-03, -1.105E-03	2.091898575	1.064812905
H7N6F1E2L1	−0.000421909	9718	9.14226E-13	−5.142E-04, -3.191E-04	1.990612673	0.993212533	−0.000358146	15363	4.08903E-11	−4.825E-04, -2.742E-04	1.802276681	0.849820507
H7N6F2E1L2	−0.000172145	9265	3.40983E-14	−2.341E-04, -1.302E-04	1.957405156	0.968942405	−0.000158469	12249	3.97476E-19	−2.233E-04, -1.323E-04	1.997725162	0.998358117
H7N6F0E3L1	−0.000673024	8675	3.53983E-16	−8.466E-04, -5.143E-04	1.941695611	0.957317055	−0.000584096	14155	6.03412E-14	−7.331E-04, -4.074E-04	1.753956647	0.810613089
A4Fa	−0.018205959	10453	1.26988E-10	−2.464E-02, -1.245E-02	1.883239859	0.913216761	−0.01737756	15542	1.00307E-10	−2.327E-02, -1.211E-02	1.788044586	0.838382711
CFa	−0.001064005	10753	8.24797E-10	−1.475E-03, -7.443E-04	1.833556463	0.874644693	−0.001161761	14094	4.24967E-14	−1.535E-03, -8.426E-04	1.929596553	0.948299235
H7N6F1E1L2	−0.000454353	10098	1.24685E-11	−5.661E-04, -3.447E-04	1.563655363	0.644922571	−0.000462898	13987	2.28621E-14	−5.780E-04, -3.497E-04	1.588369357	0.667546434
A4FE	−0.034608714	9243	2.89249E-14	−4.348E-02, -2.584E-02	1.553742606	0.635747526	−0.02954874	14875	3.23709E-12	−3.829E-02, -2.149E-02	1.446997936	0.533062864
H6N5F1E2L1	−0.012928867	9994	6.18002E-12	−1.670E-02, -9.239E-03	1.552912	0.634976077	−0.010462571	16134	1.72029E-09	−1.394E-02, -7.069E-03	1.396888031	0.482216385
TA4L	−0.009546709	9309	4.73232E-14	−1.216E-02, -7.050E-03	1.53876715	0.621774936	−0.009979695	12822	1.77467E-17	−1.238E-02, -7.632E-03	1.564958253	0.646124172
H4N3F1E1L0	−0.000378964	11514	6.56136E-08	−5.618E-04, -2.450E-04	1.527360195	0.611040331	−0.000485353	13326	4.31247E-16	−6.383E-04, -3.369E-04	1.685274019	0.752983187
A3LF	−0.102216601	9905	3.36276E-12	−1.325E-01, -7.291E-02	1.523287438	0.607188198	−0.094563735	15419	5.42458E-11	−1.223E-01, -6.702E-02	1.435787509	0.521842252
A4FS	−0.089443483	9074	8.05079E-15	−1.119E-01, -6.699E-02	1.515877628	0.600153294	−0.078844343	14357	1.89856E-13	−1.001E-01, -5.843E-02	1.432390715	0.518425072
A1F	−0.046002454	10146	1.71808E-11	−6.292E-02, -3.113E-02	1.492857151	0.578076123	−0.05080502	12888	2.71679E-17	−6.716E-02, -3.680E-02	1.554828183	0.636755164
A1SF	−0.046002454	10146	1.71808E-11	−6.292E-02, -3.113E-02	1.492857151	0.578076123	−0.05080502	12888	2.71679E-17	−6.716E-02, -3.680E-02	1.554828183	0.636755164
A4FL	−0.05447357	9028	5.65809E-15	−6.806E-02, -4.102E-02	1.492622973	0.577849796	−0.04893763	14072	3.74302E-14	−6.163E-02, -3.677E-02	1.423205939	0.509144436
A4F	−0.092475741	9329	5.48931E-14	−1.166E-01, -6.867E-02	1.490692436	0.575982627	−0.081174541	14760	1.7469E-12	−1.039E-01, -5.891E-02	1.406459449	0.492067957
A4EF	−0.092388254	9409	9.90121E-14	−1.167E-01, -6.843E-02	1.485022284	0.57048458	−0.08123373	14841	2.6995E-12	−1.043E-01, -5.862E-02	1.402145799	0.487636373
A4SF	−0.092388254	9409	9.90121E-14	−1.167E-01, -6.843E-02	1.485022284	0.57048458	−0.08123373	14841	2.6995E-12	−1.043E-01, -5.862E-02	1.402145799	0.487636373
TA4E	−0.009191054	9717	9.07802E-13	−1.186E-02, -6.718E-03	1.473756216	0.559497898	−0.009721826	13251	2.70655E-16	−1.218E-02, -7.379E-03	1.506474377	0.591176135
A1EF	−0.046093344	10268	3.84392E-11	−6.311E-02, -3.102E-02	1.47132638	0.557117311	−0.051674461	12894	2.82368E-17	−6.819E-02, -3.755E-02	1.539486008	0.622448755
A1L0F	−0.046093344	10268	3.84392E-11	−6.311E-02, -3.102E-02	1.47132638	0.557117311	−0.051674461	12894	2.82368E-17	−6.819E-02, -3.755E-02	1.539486008	0.622448755
TA4	−0.009168512	9713	8.82546E-13	−1.185E-02, -6.703E-03	1.468051514	0.553902593	−0.009744171	13229	2.35947E-16	−1.221E-02, -7.399E-03	1.502683148	0.587540838
CA4	−0.009262472	9749	1.13708E-12	−1.196E-02, -6.754E-03	1.46361774	0.549538808	−0.009868302	13217	2.18905E-16	−1.237E-02, -7.511E-03	1.500942456	0.585868667
A34F	−0.08328953	9992	6.09657E-12	−1.072E-01, -5.900E-02	1.460387177	0.546350906	−0.078418236	15204	1.81557E-11	−1.003E-01, -5.532E-02	1.398499757	0.483880003
A3EF	−0.080237836	10179	2.13909E-11	−1.039E-01, -5.616E-02	1.445343397	0.531412302	−0.075921853	15477	7.2561E-11	−9.788E-02, -5.321E-02	1.38661645	0.471568781
A3F	−0.080237836	10179	2.13909E-11	−1.039E-01, -5.616E-02	1.445343397	0.531412302	−0.075921853	15477	7.2561E-11	−9.788E-02, -5.321E-02	1.38661645	0.471568781
A3SF	−0.080237836	10179	2.13909E-11	−1.039E-01, -5.616E-02	1.445343397	0.531412302	−0.075921853	15477	7.2561E-11	−9.788E-02, -5.321E-02	1.38661645	0.471568781
H3N4F0E0L0	−0.000158411	10870	1.67315E-09	−2.208E-04, -8.045E-05	1.441911762	0.527982882	−0.000173979	14105	4.5277E-14	−2.302E-04, -1.375E-04	1.564080738	0.645314986
A4LF	−0.090054475	9988	5.93297E-12	−1.163E-01, -6.493E-02	1.433148107	0.519187711	−0.07783696	15762	2.94984E-10	−1.021E-01, -5.383E-02	1.351347835	0.43439907
A2S0G	0.140194851	30024	5.13958E-36	1.212E-01, 1.594E-01	0.756133937	0.403286287	0.116166115	39404	2.65068E-30	9.818E-02, 1.342E-01	0.790332459	0.339468434
A2FS0G	0.136941696	29754	1.34452E-34	1.180E-01, 1.563E-01	0.755659732	0.404191349	0.113087239	38996	8.62464E-29	9.508E-02, 1.314E-01	0.790315386	0.339499599
H6N5F0E2L0	0.002231585	26763	7.23795E-21	1.808E-03, 2.657E-03	0.719189514	0.475556108	0.002374427	37922	5.29388E-25	1.968E-03, 2.798E-03	0.707015758	0.500185725
H5N4F0E1L0	0.023719036	27158	1.7924E-22	1.980E-02, 2.757E-02	0.710562245	0.49296706	0.018094108	36292	8.68547E-20	1.494E-02, 2.091E-02	0.783538872	0.351923245
H5N4F0E0L2	0.000602187	27757	4.98381E-25	5.061E-04, 6.894E-04	0.697065025	0.520634851	0.000433998	36382	4.6518E-20	3.595E-04, 5.386E-04	0.765562088	0.385408708
H6N5F0E1L1	0.003485357	28194	5.51408E-27	2.939E-03, 3.985E-03	0.679944779	0.55651051	0.003030007	37651	4.31926E-24	2.484E-03, 3.540E-03	0.703024141	0.508353864
A2Fa	0.000244241	28121	1.18469E-26	1.841E-04, 2.385E-04	0.677663015	0.561360059	0.00019451	37712	2.70246E-24	1.355E-04, 2.072E-04	0.772706325	0.372007887
H5N4F2E0L0	0.000161745	27406	1.63237E-23	1.563E-04, 1.758E-04	0.670475145	0.576744244	0.000134029	36606	9.64265E-21	1.058E-04, 1.564E-04	0.774074214	0.369456204
H5N4F0E0L0	0.001038368	28415	5.28256E-28	8.589E-04, 1.185E-03	0.510165943	0.970961502	0.000723915	37191	1.38707E-22	6.036E-04, 8.483E-04	0.690880212	0.533492503

See also Table S3.

**Figure 4:**
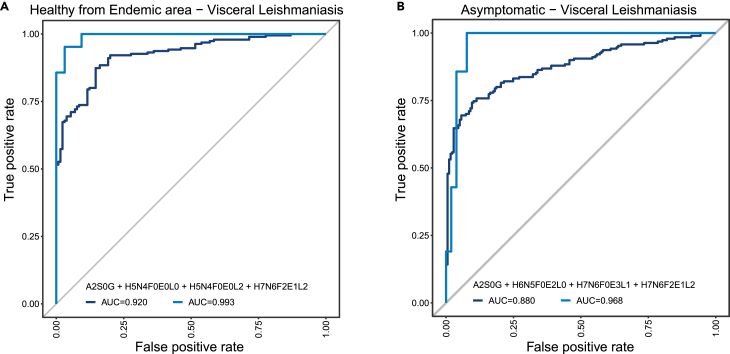
VL can be detected using a panel of four glycosylation traits ROC analysis showing models build on traits as determined by automated feature selection using the SES algorithm of the MXM R-package. A 10-fold cross-validation procedure was used to assess the strength of the prediction models. The models were then validated using an independent dataset. The relevant analysis values are given in Table S4. (A and B) Endemic Controls vs. Visceral Leishmaniasis and B) Asymptomatic vs. Visceral Leishmaniasis. Dark blue line: cross-validated model as determined by the SES algorithm. Light blue line: same model applied to the validation set.

For the distinctive power of VL against the other samples, ROC analysis using automated feature selection from the shared significant glycans and glycosylation traits on the discovery set yielded a four-parameter model (A2S0G + H5N4F0E0L0 + H5N4F0E0L2 + H7N6F2E1L2) against the healthy control group as well as a four-parameter model (A2S0G + H6N5F0E2L0 + H7N6F0E3L1 + H7N6F2E1L2) for the asymptomatic group. The predictive power of these cross-validated models is very good, with respective AUCs of 0.92 and 0.88, sensitivities of 0.854 and 0.859 and specificities of 0.874 and 0.758. Applying these models to the validation set even outperformed the discovery set with AUCs of 0.99 and 0.97 respectively (Figures 4A and 4B).

### *N*-glycome is not influenced by the severity of VL

To better understand underlying processes of active VL and progression of disease, we divided the VL group of the combined discovery and validation sets in two sub-groups: uncomplicated (U-VL) and complicated with clinical manifestations (C-VL). We did so, because the validation set only contained two complicated cases, which makes the set unsuitable for validation. The latter group included patients with additional therapy, hemorrhage or increased risk of death. The data as depicted in the Volcano Plot (Figure S4C) indicate that none of the glycans or glycosylation traits are significantly different between these groups (Table S3G). Even in the case of experiment-wise multiple testing correction (α = 0.05/226 = 2.2124e-4) instead of study-wide multiple testing correction, only one glycan (H5N5F0E1L0) would be significantly different between these groups with an absolute log_2_(fold-change) of at least 0.3 (decrease in this case; −0.515) and possibly another one with an absolute log_2_(fold-change) of just below 0.3 (H6N5F1E1L0; −0.298). This result is corroborated by ROC analysis (Figure S5C). The obtained single parameter model (H6N5F1E1L0) was poorly predictive, with an AUC of 0.60. Of interest, H5N5F0E1L0 was not selected as a discriminator in this ROC analysis.

### *N*-glycome reverts to base levels after VL treatment

**Figure 5:**
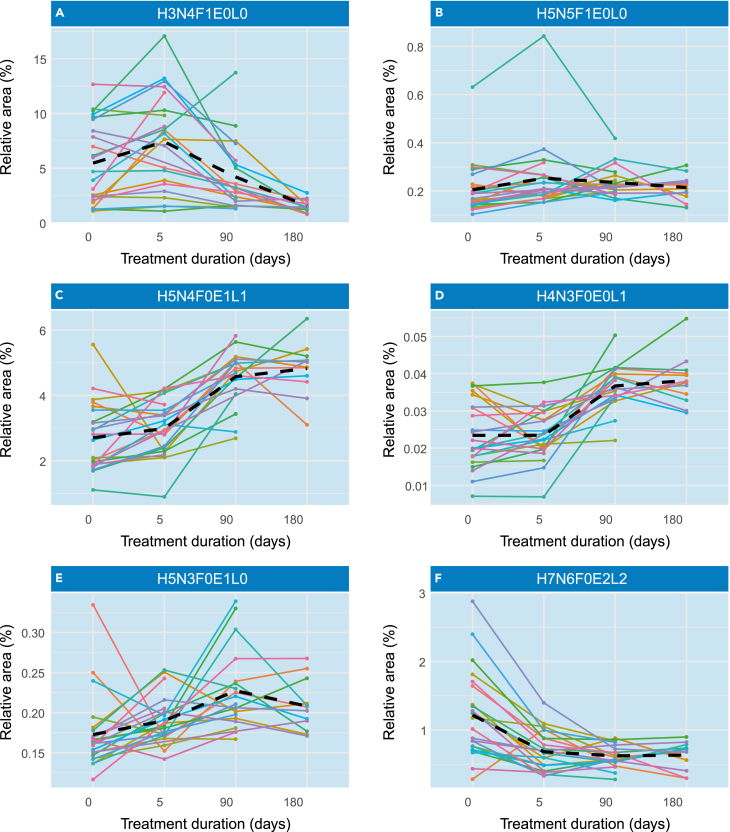
Changes in the *N*-glycome of VL patients are reverted on treatment Changes in the *N*-glycome signature in plasma samples from patients with visceral leishmaniasis after beginning of treatment. The spaghetti plots show the same representative set of glycans as depicted in Figure 2 and each line represents the *N*-glycome signature from one individual in different time points after the onset of treatment. The black dashed line represents the mean between individuals. (A–F) H3N4F1E0L0, B) H5N5F1E0L0, C) H5N4F0E1L1, D) H4N3F0E0L1, E) H5N3F0E1L0, F) H7N6F0E2L2. H = hexose, N=N-acetylhexosamine, F = deoxyhexose (fucose), L = lactonized N-acetylneuraminic acid (α2,3-linked), E = ethyl esterified N-acetylneuraminic acid (α2,6-linked).

To evaluate the glycosylation changes on treatment and recovery from disease we assessed the difference in glycan levels in patients’ plasma samples at day 0, 5, 90 and 180 after onset of treatment. The declining number of samples over time was caused by patients unfortunately not returning to the hospital at the indicated timepoint. Therefore, the number of samples was too low to perform statistical analysis. However, several trends were observed. On treatment, most of the glycosylation patterns returned to the situation as observed in healthy controls. Fucosylation in general (Figures 5A, 5B, 5C, and 5F) and sialylation on the tetra-antennary glycans (Figure 5G) decreased over time whereas α2,3- and α2,6-linked sialylation on diantennary glycans increased (Figures 5D and 5E). Bisection of fucosylated diantennary structures showed a decrease in VL patients, increasing over time on recovery.

## Discussion

TSNG analysis provided a strong signature that allowed to differentiate VL from both asymptomatic and endemic controls with very high sensitivity and specificity (AUCs of 0.88 and 0.92 for ASYMP and EC, respectively). Given the invasive nature of current diagnostic approaches for VL, blood glycome analysis may provide a promising approach for further evaluation and possible implementation in VL diagnosis. Blood glycome analysis can be performed from dried blood spots and provides a glycomic signature that is very similar to the one obtained by glycomic analysis of serum or plasma.^44^ Hence, further research into dried bloodspot glycome analysis from clinical cohorts of active VL and conditions with similar clinical presentation is warranted to evaluate its potential for complementing or replacing current invasive diagnostic procedures. In addition, for clinical implementation, blood glycome analysis will have to be transferred from high-end mass spectrometry to e.g., a microtiter plate assay.^31^^,^^41^^,^^45^

In our study we included both healthy controls from endemic as well as from non-endemic areas. The analysis indicated differences between these two groups that pointed toward the involvement of immunoglobulins. However, most of these differences could not be validated. As these differences are clearly not VL-induced, they might be caused by a difference in lifestyle and/or environment, as previously demonstrated.^46^ People living in endemic areas might experience higher exposure to pathogens in general causing higher levels of proteins involved in protection against them. Because the endemic controls are more closely related to asymptomatic and active VL cases and to exclude bias introduced by demographic differences, we decided to exclude the non-endemic controls from further comparisons. Following this reasoning we would like to stress that the discussion described hereafter should be considered as specific for the population under study as well as the parasite species.

Visceral leishmaniasis, when in its chronic course, presents clinical signs like those found in systemic erythematous lupus,^47^ such as enlarged liver and spleen, and pancytopenia^48^ and the clinical status of the disease is directly associated with a systemic inflammatory response.^49^^,^^50^ It is known that the progression of liver diseases is associated with specific glycosylation alterations in serum proteins. For instance, in hepatocellular carcinoma, a disease with high risk for hepatic injury and inflammation, there is an increase in levels of core fucosylation.^51^ The serum *N*-glycome from patients infected with *Pseudomonas aeruginosa*, an opportunistic gram-negative pathogen closely associated with cystic fibrosis affecting lung and liver, shows increased levels of core fucosylation (as well as decreased levels of sialylation, which will be discussed later).^52^ Core fucosylation levels are also increased in advanced stages of pancreatic cancer, however the authors of that study suggested that the biological function seemed to be related to the disease itself and not to the inflammation.^53^ Similar glycan profiles have been described for (auto)inflammatory diseases such as rheumatoid arthritis^54^ and Crohn’s disease,^30^^,^^55^ in which fucosylation of a tri-antennary structure, indicated as an inflammatory marker, was elevated. To this extent, our results of increased fucosylation, both on the core and on the antennas, corroborate with all these observations indicating a large involvement of the inflammatory pathways. As has been shown previously, *L. infantum* leads to an elevation of circulating immunoglobulins in VL patients.^56^^,^^57^ Of interest, patients with dengue virus^58^ and COVID-19^59^ had reduced levels of core fucosylation on disease-specific IgGs. This reduction was specifically correlated with enveloped viruses. The biological function of core fucose reduction is associated with an excessive activation of FcγRIIIa, which leads to an overreaction by the immune cells, increased antibody-dependent cellular cytotoxicity (ADCC) and cytokine storms.^60^ As we observed the opposite response with regard to fucosylation in TSNG, and a similar increase in fucosylation was observed in total IgG of VL patients,^38^ it would be interesting to investigate glycan profiles of *Leishmania*-specific IgG, especially of severe cases because these proinflammatory responses are also observed in patients presenting with severe VL^49^ and they are possibly associated with a decreased fucosylation in specific IgGs, thus explaining this phenomenon. It is noteworthy that, compared to uninfected controls or asymptomatic individuals, in patients presenting with active VL the transcriptional profiles of genes expressed in peripheral blood leukocytes annotated into processes of leukocyte chemotaxis, neutrophil activation and B cell receptor activation were down-regulated whereas, on the other hand, up-regulated genes were mainly enriched into the network process of NK cell cytotoxicity,^61^ suggesting the involvement of FcγRIIIa.

Besides increased fucosylation, other changes in inflammatory glycosylation patterns include changes in bisection, galactosylation and sialylation. To start with bisection, our results are in accordance with a study on thrombocytopenia where treatment of the disease resulted in an increase in bisection, suggesting reduced bisection on development of symptoms.^62^ On the other hand, bisection was significantly higher in meningitis patients presenting with the most severe clinical outcomes.^63^ Bisection also discriminates between pathogens that cause bacteremia,^52^ possibly reflecting differences in their microbial-associated molecular patterns and respective strengths in activating innate immune signaling pathways.

With regard to galactosylation and sialylation, our results are in line with previous findings in other diseases with inflammatory profiles. A decrease of total IgG1 sialylation, galactosylation and bisection and increase of fucosylation was also observed in relapsing vasculitis patients with higher anti-neutrophil cytoplasmic antibody levels.^64^ The previous work on the glycosylation patterns of IgG molecules of VL patients also showed a reduction of bisection, sialylation and galactosylation of, especially the Fc portion of IgG subclasses in VL patients and asymptomatic individuals, although the decrease in this last group was less pronounced, consistent with our findings for TSNG. Of interest, the decrease of galactosylation observed in our data was mainly caused by the effect on diantennary glycans and the decrease of sialylation as a result of decreased α2,6-linked sialic acids. These results are in accordance with the fact that Immunoglobulins comprise a large group of proteins in the human body and the Fc portion of IgGs carries diantennary glycans.^38^ When present, sialylation on IgG is almost exclusively α2,6-linked. Both observations indicate a strong relation with IgG glycosylation.^65^ Furthermore, a connection has been proposed between decreased galactosylation and both age and inflammatory diseases.^36^ Because the mean age of the group with complicated cases is relatively low (23 years), and the proinflammatory effect of IgG is associated with an increase in agalactosylated structures,^66^ we hypothesize that the observed change in galactosylation is a result of the inflammatory profile of VL.

The above-described results all point toward a large contribution of immunoglobulins and their glycosylation to blood glycosylation signatures. With regard to hypergammaglobulinemia, one may speculate that specific antibody glycoforms may contribute to inflammation and regulate immunity (in much the same way that overall levels of serum cytokines determine immune profiles and clinical outcomes). Furthermore, there was indication in the results for some disease-specific changes as well. An increase in antennarity has been observed, especially the tetra-antennary structures. In contradiction with the general decrease in galactosylation and sialylation, these glycosylation traits showed an increase on tetra-antennary glycans as well, which for sialylation was not linkage specific. The question remains if a change in the number of antennae is indicative of a disease-specific phenomenon, as such processes also have been identified in patients exhibiting inflammation of different origin. For instance, after inflammation-mediated processing, the carbohydrate structures of various inflammatory glycoproteins shift from high-mannose type to di-, tri- and tetra-antennary glycans.^67^ In addition, it has been reported that the glycans on alpha-1-acid glycoprotein (AGP), one of the major acute phase proteins in humans, are modified during an acute phase response, from bito tri- and tetra-antennary branching with increased fucosylation and sialylation. Similar modification of AGP with increased branching of the glycan structures has also been reported in some inflammatory diseases such as asthma and rheumatoid arthritis.^68^ Of interest, in patients with liver cirrhosis AGP fucosylation was higher. An increase in the levels of both α2,3- and α2,6-sialylation has been reported as general inflammatory markers shared by many diseases.^69^ For example, in autoimmune diseases, inflammatory bowel diseases and acute inflammation the higher branching with increased sialylation has been described.^30^

The presence of sialic acid has been reported both in the promastigote and in the amastigote form of *Leishmania*. However, the detection of these sugar molecules in the parasite is still questionable, since the biosynthetic machinery of the parasite to acquire sialic acid is not well understood, and most of the studies are performed in *Leishmania donovani*, without considering the fact that *Leishmania* species are heterogeneous with regard to molecules and virulence. Despite that, it has been shown that *L. donovani* can adsorb sialoglycans under various stimuli in the environment to possibly compensate for deficiency of sialic acid^70^ and, as reviewed by Cavalcante et al. and Colli, to evade host immunity.^71^^,^^72^ Such mechanisms may thus explain the decrease in sialic acid-containing glycans seen on proteins in patients presenting with VL. A similar hypothesis has been stated for individuals infected with *P. aeruginosa*.^73^ Here, the bacterium was shown to express a sialidase that cleaves sialic acid residues from the host’s glycoproteins thereby facilitating adherence to host cells, which was inhibited by sialylated glycans on the host cell surface. Khatua et al. also reported on *P. aeruginosa* capable of absorbing host α2,3- and α2,6-sialoglycoproteins, thereby reducing neutrophil activity and increasing survival of *P. aeruginosa*.^74^

Our results also may finally provide the basis of a mechanism to explain earlier findings, that sera from VL patients contain factors that enhance three-to 5-fold the lysis of amastigotes of *L. donovani* by the alternative pathway of complement when compared to normal human serum.^75^ Another of the observations made at the onset of leishmaniasis involves ending the lytic route.^76^ The parasite-specific IgG antibody induces lysis of *Leishmania* (and other trypanosomatids).^77^ Sialic acids are crucial for the protection of the parasite against attack by the host complement system,^78^ similar to what occurs, for instance, with *Neisseria gonorrhoeae*, which is protected from activated mannose-binding lectin (MBL) complement and death by sialylation both in epimastigote and trypomastigotes forms of the parasite, preventing the binding of lytic anti-galactose antibodies.^79^ It is interesting to note that if the success of *Leishmania* infection in the cell depends on sialic acid, the greater the parasite’s ability to adsorb it, the greater the parasite’s proliferation and, probably, a greater damage to the host. Unfortunately, so far, the deeper analysis of glycosylation in relation to severity of the infection did not elucidate such close relationships and in this context, antigen-specific antibodies must be examined. Further possible mechanisms that might be the basis of the associations of TSNG profiles with the outcomes of infections with *L. infantum* seen in this study are the resulting interactions of *L. infantum*-specific antibodies with the several receptors on phagocytes that mediate this process (reviewed in^80^), as well as the pathogen escape mechanisms that are possibly mediated by the receptors for IgG Fcs present on many pathogens, including trypanosomatid pathogens.^81^

Although not significant, the two most promising glycans with a relation toward disease severity were already slightly reduced in asymptomatic cases and significantly reduced in VL patients overall. If we plot the two severity groups separately next to the asymptomatic and control groups (Figures S5A and S5B) it can be observed that there is a clear downward trend of H5N5F0E1L0, decreasing with severity and the reduction of H6N5F1E1L0 can be almost entirely contributed to severe disease. The mean for this glycan is similar between the asymptomatic and the uncomplicated group, already slightly reduced compared to the healthy controls and lowest in the complicated VL group. Therefore, we dare to hypothesize that these glycans are influenced by the severity of the disease. Unfortunately, this change is not sufficiently pronounced to be used as a predictive marker as is shown with the ROC analysis. The biological meaning of this still remains unclear. Furthermore, it is notable that all the changes described above seem to revert on treatment.

Our research was driven by two main questions. One of these questions involved the predictability or early diagnosis of VL infection and severity. Early detection in the form of asymptomatic cases appears not to be possible using the methods described herein as (univariate) analysis did not yield enough consistent and replicated differences. Nonetheless, it would be insightful to undertake a longitudinal study in an endemic area that follows individuals bearing asymptomatic infections with *L. infantum*.

Our second question addressed the possibility whether the glycan analysis had potential for diagnosis as a substitute for the current invasive techniques. ROC analysis revealed that good models could be built for the discrimination of VL cases against both healthy controls and asymptomatic cases. In both cases, this resulted in a four-parameter model. Of interest, two parameters are shared between the models. Because the model comparing VL against healthy controls has the most logical medical meaning when used as a diagnostic tool, we feel that the four parameter EC-VL model (A2S0G + H5N4F0E0L0 + H5N4F0E0L2 + H7N6F2E1L2) is the final one, although the ASYMP-VL model performed slightly better in the validation set. This might be caused by a different sample distribution or the different diagnostic standard. Ongoing investigation using new samples will be required to enforce our model. Glycan analysis might be a good replacement of current methods to detect VL, because it is less invasive for the patient. However, the question remains if the model is disease-specific or, in view of the findings discussed above, a representation of general disease phenomena. Therefore, its diagnostic potential should be confirmed by cross validation with more samples from patients presenting with VL and relevant clinical controls (e.g., systemic inflammatory diseases that affect the liver, such as lupus,^47^ and neoplasias involving bone marrow such as multiple myeloma,^82^ etc.). It is worth mentioning at this point that VL can be confused with lupus and lymphoma and this can be fatal for the VL patient if not treated promptly because of this confusion. These appropriate controls should also include patients free from VL but suffering from diseases that can occur as concomitant conditions with VL, such as HIV, leprosy and Chagas Disease to exclude cross-reactivity; more research is needed in which multiple diseases involving similar phenotypes are compared to each other with respect to predictability.

It is also relevant to highlight that even after successful treatment and in case of immunosuppression, recurrence of VL may occur if there are viable parasites.^83^ Besides that, there are VL cases with presence of antibodies beyond cure and there are some patients who are not able to produce sufficient antibodies, thus limiting the diagnosis of relapse or the prediction of cure by antibody-based tests such as rK39 rapid strip immunochromatographic test and ELISA.^24^ In this sense, our results indicate that the glycomic signatures reflect treatment response and Total Plasma *N*-Glycome (TPNG) analysis (or TSNG analysis) could be used to monitor treatment success and eventually detect relapses. Finally, VL caused by *L. infantum* is a zoonosis with dogs being the reservoir. However, although there is no scientific evidence supporting culling of seropositive dogs to reduce the incidence of VL, asymptomatic dogs present low parasitism whereas symptomatic dogs are associated with high parasite load in various tissues such as skin, bone marrow and spleen.^84^^,^^85^ The diagnosis in dogs faces the same issues as it does in humans, and it may also be worth examining TSNGs in this host to better define the *de facto* animal reservoirs for humans in this species.

In conclusion, TSNG could become a tool for detecting VL with high sensitivity and specificity, because the distinction of active VL from healthy controls or asymptomatic cases was clear, whereas between complicated and uncomplicated VL the TSNG signature appears to be largely the same. As for the individuals presenting with asymptomatic infections with *L. infantum*, although some structures seem to be slightly decreased in this group (ASYMP) compared to the controls (EC), their change has not sufficient statistical power to be used as a biological marker of infection nor were indications present of a subgroup at risk to evolve to VL. The changes in the *N*-glycome signature are associated with increased core- and antenna fucosylation and reduction of bisection, galactosylation and sialylation of especially diantennary glycans which has already been described for other diseases with an inflammatory profile, mainly the ones associated with liver injury. In addition, the increase in antennas as well as the increase in galactosylation and sialylation of these larger glycans may be associated with inflammation in active VL. Therewith, the overall glycan profile of VL has an inflammatory signature, which aids in understanding the pathogenesis of the disease, indicating a potential target for treatment and relapse detection if better explored. Until now, a disease specific signature could not be identified, which requires additional research.

### Limitations of the study

It is noteworthy that infections were characterized with different strategies for the 2013 discovery and 2018 validation sets because of a change in regulatory standards: the Montenegro skin test reagent employed to elicit parasite-specific delayed hypersensitivity skin reactions, one of the approaches for diagnosis of infections with *L. infantum*, was discontinued after 2013. This required the development of a new assay based on the release of select cytokines by blood leukocytes.^26^ The difference in assignment strategies may have caused a difference in assignment accuracy, which may in part explain why the validation outperformed the discovery. A second contribution to this outperformance may be because of differences in the sample distribution for EC versus VL in the discovery set (2:3) versus validation set (3:2). This reflects, however, the expected fluctuations seen in the incidence of VL by public health surveillance.^86^

Furthermore, although the observed differences between VL and healthy or asymptomatic controls are striking, they do not elucidate disease specificity. To that purpose, further comparison with unrelated diseases with similar phenotypes or regularly observed co-infections is required.

## STAR★Methods

### Key resources table

**Table:** 

REAGENT or RESOURCE	SOURCE	IDENTIFIER
**Biological samples**		

Sera from individuals presenting with active visceral leishmaniasis confirmed by presence of amastigote forms in bone marrow aspirates stained by Giemsa, and confirmed by isolating parasites in cultures of the bone marrow aspirates in NNN medium.	Patients hospitalized at the Nathan Portela Institute of Tropical Medicine, Federal University of Piaui or at the University Hospital of the Federal University of Sergipe in 2012-2013; Gardinassi et al.^38^	N/A
Sera from individuals presenting with active visceral leishmaniasis confirmed by presence of amastigote forms in bone marrow aspirates stained by Giemsa, and confirmed by isolating parasites in cultures of the bone marrow aspirates in NNN medium.	Patients hospitalized at the Nathan Portela Institute of Tropical Medicine, Federal University of Piaui, 2018; Porcino et al.^26^	N/A
Sera from individuals presenting with asymptomatic infections or no infection (uninfected endemic controls)	Contacts (neighbors and household members) of VL patients, 2012-2013; Gardinassi et al.^38^	N/A
Sera from individuals presenting with asymptomatic infections or no infection (uninfected endemic controls)	Contacts (neighbors and household members) of VL patients, 2018; Porcino et al.^26^	N/A

**Chemicals, peptides, and recombinant proteins**		

Soluble antigen of *L. infantum* (SLA) for diagnostic serology and cytokine release assay	Gardinassi et al.^38^; Porcino et al.^26^	N/A
PNGaseF	Merck, Darmstadt, Germany	Cat#11365177001

**Critical commercial assays**		

Kalazar Detect Rapid	InBIOS International, Seattle, WA, USA	Cat#INS025
OnSite Leishmania IgG/IgM Combo test	CTK Biotech,San Diego, CA, USA	Cat#R0122S
BD Cytometric Bead Array Human Flex Set IP-10 human	Becton Dickinson Biosciences, USA	Cat#558280
BD Cytometric Bead Array Human Flex Set Anti-MIG human E8	Becton Dickinson Biosciences, USA	Cat#558286
Human Soluble Protein Buffer Kit CBA Flex Set	Becton Dickinson Biosciences, USA	Cat#558264

**Deposited data**		

RAW, analyzed and meta data	https://glycopost.glycosmos.org/	GPST000313

**Software and algorithms**		

Rstudio	Rstudio Team^91^	https://posit.co/
MXM R-package	Lagani et al.^92^	https://cran.r-project.org/package=MXM
MassyTools	Jansen et al.^90^	https://github.com/Tarskin/MassyTools

**Other**		

Hamilton STAR and STARplus robotic system	Hamilton	https://www.hamiltoncompany.com/automated-liquid-handling
Bruker solariX 15T FT-ICR-MS	Bruker	https://www.bruker.com/en/products-and-solutions/mass-spectrometry/mrms/solarix.html

### Resource availability

#### Lead contact

Further information and requests for resources and reagents should be directed to and will be fulfilled by the lead contact, Isabel Kinney Ferreira de Miranda Santos (imsantos@fmrp.usp.br).

#### Materials availability

This study did not generate new unique reagents.

### Experimental model and study participant details

This work was limited to human subjects. The total number of 661 participants was divided into a 338:264 ratio female:male (for 59 participants sex was not registered). Throughout the text, when sex is mentioned, the term sex assigned at birth is meant. Age ranged from 2 to 86 years. All participants originated from the Teresina or Aracajú region of Brazil. Other racial or ethnic information was not registered. More details are presented in Table 1. All available metadata per patient is also included in Table S5. This study has been approved by the Research Ethics Committee of the Clinics Hospital of the Ribeirão Preto School of Medicine of the University of São Paulo, by signature of the responsible researcher described by protocol 2347/2012, or Ethics Presentation Certificate number 67213017.0.0000.5440 and opinion number 2.101.755. Furthermore, the certificate number for authorization of research at the Institute of Tropical Diseases Natan Portela is AA.901.1.009518/17-65. The methods applied in this study were performed according to the approved guidelines, and an informed consent was obtained in all cases.

### Method details

#### Patient recruitment and sample collection

For this study, samples were collected at two different timepoints, the discovery set in 2012-2013 and the validation set in 2018. Serum samples were collected from patients with active Visceral Leishmaniasis (VL: n=211), hospitalized at the Institute of Tropical Diseases Natan Portela (Teresina, Piauí, Brazil, discovery and validation), or at the University Hospital, UFS, Aracajú-SE (discovery only). Positive diagnosis was confirmed by presence of the amastigote forms of *Leishmania infantum* in bone marrow aspirate using Giemsa staining, and cell culture in NNN medium. Additionally, serum samples were collected from individuals with asymptomatic infections (ASYMP: n=229), healthy individuals living in an endemic area in Teresina city or Aracajú city, Brazil (EC; n=162), and healthy individuals living in a non-endemic area (NC: n=59) in Ribeirão Preto city, Brazil. In order to classify asymptomatic, positive (at least in one of the tests described below), and healthy individuals regarding VL (negative in all tests applied), all samples were tested to detect the presence of antibodies against *Leishmania* by ELISA, the Kalazar *Detect*™ Immunochromatographic test and the presence of the parasite in the blood by qPCR,^26^ with a positive Montenegro skin test (MST^+^),^38^ or the production of cytokine profiles upon stimulation of peripheral blood leukocytes with an antigen extract prepared from promastigotes of *L. infantum*.^26^ Due to a change in regulatory standards the Montenegro skin test was no longer permitted after 2013 by the Brazilian Health Ministry and was therefore only applied to the discovery set.

In this study we assessed the categories of clinical severity of 131 patients, which were classified in uncomplicated (U-VL: n=49) and complicated with required additional therapy or with hemorrhage and increased risk of death (C-VL: n=82).

Plasma samples from VL patients (n=24) monitored for 180 days after the beginning of treatment were collected at day 0 (n=23), 5 (n=22), 90 (n=20) and 180 (n=12).

#### Chemicals and reagents

Analytical grade ethanol, sodium dodecyl sulphate (SDS) and trifluoroacetic acid (TFA) were purchased from Merck (Darmstadt, Germany). Disodium hydrogen phosphate dihydrate, potassium dihydrogen phosphate sodium chloride, 85% phosphoric acid, 50% sodium hydroxide, nonidet P-40 substitute (NP-40), 1-hydroxybenzotriazole 97% (HOBt) and super-DHB (9:1 mixture of 2,5-dihydroxybenzoic acid and 2-hydroxy-5-methoxybenzoic acid, sDHB) were obtained from Sigma-Aldrich (Steinheim, Germany). 1-Ethyl-3-(3-(dimethylamino)propyl) carbodiimide (EDC) hydrochloride was purchased from Fluorochem (Hadfield, UK), whereas recombinant peptide-N-glycosidase F (PNGase F) was purchased from Merck (Darmstadt, Germany). HPLC-grade acetonitrile (ACN) was obtained from Biosolve (Valkenswaard, The Netherlands) and ultrapure water (Milli-Q or MQ, resistance ≥18 MΩ) was generated from a Q-Gard 2 system (Millipore, Amsterdam, The Netherlands). The cotton used for HILIC purification was obtained from Pipoos (Utrecht, The Netherlands).

#### *N*-glycan release

All clinical samples were distributed over 96-well plates in a randomized manner, together with technical replicates of a commercially available pooled plasma standard (Visucon-F: Affinity Biologicals (Ancaster, ON, Canada) and a pooled serum from clinical samples randomly chosen, to monitor the quality of the glycomic sample preparation and measurements. Multiple instances of Phosphate Buffered Saline (PBS) were also included as blanks. Glycans were released from proteins as described previously.^40^ After denaturation of 6 μL of serum with 12 μL 2% SDS at 60°C for 10 min, *N*-glycans were enzymatically released from serum glycoproteins using a fresh releasing mixture which contained 6 μL of 4% NP-40, 6 μL of acidified PBS (100 mM phosphoric acid in 5X PBS) and 0.6 μL of PNGase F for each sample and incubated overnight at 37°C.

#### Derivatization, purification and preparation for mass spectrometry analysis

The automated sample preparation consists of derivatization, hydrophilic interaction liquid chromatography (HILIC) purification, and MALDI-target plate spotting which was performed using an automated liquid handling platform.^40^ Ethyl esterification, for stabilization and linkage-specific derivation of sialic acids, was performed with freshly prepared chemicals and solutions. Therefore, 2 μL of the released glycan samples was added to 40 μL of ethyl esterification reagent, which consisted of 0.25 M EDC and 0.25 M HOBt dissolved in 100% ethanol, followed by incubation for 1 h at 37°C.^88^ Subsequently, 40 μL of acetonitrile was added. For the purification of the *N*-glycans, in-house assembled cotton HILIC microtips were used (approx. 3 mm or 180 μg cotton thread per tip). The tips were pre-washed with MQ water and 85% acetonitrile. Then, the glycans were bound to the cotton by pipetting the samples up and down 20 times. The tips were washed with 85% acetonitrile with 1% TFA followed by 85% acetonitrile and eluted in 20 μL MQ water. Subsequently, 7 μL of the purified sample plus 7 μL of sDHB matrix (2.5 mg/mL in 50% ACN with 0.1 mM NaOH)^89^ was premixed in a 384-well plate. Then, 2 μL of the mixture was spotted onto a MALDI target plate (800/384 MTP AnchorChip, Bruker Daltonics, Bremen, Germany), and after air-drying the spots were measured by MALDI-FTICR-MS.

#### MALDI-FTICR-MS and pre-processing of mass spectrometry data

The analysis was performed using matrix assisted laser desorption ionization – Fourier-transform ion cyclotron resonance – mass spectrometry (MALDI-FTICR-MS) on a Bruker 15T solariX XR FTICR mass spectrometer equipped with a CombiSource and a ParaCell (Bruker Daltonics). The system was controlled by ftms Control version 2.1.0 and spectra in a *m/z*-range from 1011.40 to 5000.00 with about 1.7 million datapoints per spectrum were recorded. Each single spectrum was generated from ten scans of 200 laser shots per raster point within the sample spot. DataAnalysis 5.0 SR1 (build 203.2.3586, Bruker Daltonics) was used to visualize, calibrate (Table S1), and export MALDI-FTICR-MS spectra into *xy* file format, which is compatible with the software for further processing.

#### Data processing

The *xy* text file format of the spectra and a list with 116 glycan compositions (including two dummy glycans) (Table S1) were used to extract peak areas, associated with parameters for glycan relative quantification and data quality assessment by MassyTools software version 1.0.2-alpha build 180703b^90^ The extraction window ranged from 0.00719 to 0.08921 according to the formula ‘(0.00003 ∗ "*m/z*") - 0.02690’, in order to accommodate for the increasing peak width with larger *m/z* values. The formula was obtained from the linear trendline derived from manual peak width measurements (full width at half maximum). To exclude poor quality spectra, the total intensity of a spectrum, the fraction of analyte area (minus background area) above signal to noise (S/N) 9, and the fraction of spectrum in analytes should be higher than the mean of these parameters minus three times the standard deviation. Each of these parameters was evaluated per biological group. The analyte curation was performed based on the following quality criteria: S/N should be greater than 9, isotopic pattern quality (IPQ) less than 0.2 and absolute PPM-error lower than 10 for at least 25% of all spectra per biological group. Areas of all curated glycans were normalized to the sum of these areas per spectrum and R-Studio software was used to calculate glycosylation traits (Table S2).^35^

### Quantification and statistical analysis

Analyses were performed in the R programming language version 4.1.0 (R Foundation for Statistical Computing, Vienna, Austria) and R-Studio software version 1.4.1106 (RStudio, Boston, MA).^91^ To monitor the quality of the sample preparation, repeatability was tested using the Visucon standards and pooled serum samples, by analyzing the 25 most abundant glycans.

Principal Component Analysis (PCA) on the full dataset was performed to investigate if the four distinct groups of interest could be distinguished based on the respective *N*-glycome signatures. To do so, values were log_10_ transformed and UV-scaled before PCA analysis was applied. To further evaluate the differences between the groups, the means of each individual glycan and glycosylation trait were compared using a Kruskal-Wallis test with, in case of significance, an additional post-hoc Dunn’s test. The biological significance was then further evaluated by investigating the fold change in a group-by-group comparison using a Wilcoxon rank sum test on the discovery set and illustrated in a Volcano Plot. This same test was also applied to a severity group comparison (U-VL vs. C-VL). In significance testing a study-wide significance was set at α = 5.0e-5. This value was obtained by using a default α of 0.05, Bonferroni corrected by the study-wide number of tests performed (73 glycans + 153 glycosylation traits times one Kruskal-Wallis test plus four Wilcoxon tests per trait = 1130 tests: 0.05/1130 = 4.4248e-5 ≈ 5.0e-5). The log_2_(fold-change) cut-off for the Volcano Plots was set at 0.3. Then these tests were applied to the validation set using an alpha of 0.05. Glycans and traits matching those in the discovery set with both significance and sufficient fold change were used as input for parameter selection in Receiver Operating Characteristics (ROC) analysis. Such analysis was performed in a group-by-group comparison for the comparisons EC-VL and ASYMP-VL on the discovery set and for the severity groups Uncomplicated – Complicated on the total set, since the validation set would only contain two complicated cases. Feature selection was achieved using the SES algorithm of the MXM R-package^92^ applied to log_10_-normalized and scaled data on the remaining glycans and traits from the Wilcoxon tests. This algorithm could in theory output multiple sets of covariates for a single comparison. For each of these sets of covariates a general logistic model was calculated and the one with the lowest p-value for a Chi-square test was used in a ten-fold cross validation procedure. In each iteration of the cross-validation cycle the SES algorithm was applied again with the same selection procedure for covariates. From this cross-validation procedure, the Area Under the Curve (AUC) was calculated. As an independent validation the resulting models were applied to the validation set and a ROC curve was created.

The number of samples used for the follow-up after treatment was too low for statistical analysis. Therefore, only observed trends are discussed.

## Data Availability

• Raw mass spectrometry data in the form of .xy files have been deposited at GlycoPost^87^ and are publicly available as of the date of publication. Accession numbers are listed in the key resources table. This repository also contains the relevant metadata as well as the final curated data (Table S5) used in this manuscript. • This paper does not report original code. • Any additional information required to reanalyze the data reported in this work paper is available from the lead contact upon request.

## Supplemental information

Document S1. Figures S1–S8

Table S1. Analyte list (including two dummy glycans) and relevant properties, related to STAR Methods Analyte compositions with (theoretical) *m/z*-values and extraction windows. All species are calculated as [M+Na]+. Calibrants used for internal calibration by MassyTools are colored red. The most abundant isotope of the isotopic envelope was used for calibration, in the case that the second isotope is the most abundant it is indicated by (i2). H = hexose; N = N-acetylhexosamine; F = deoxyhexose (fucose); L = lactonized N-acetylneuraminic acid (α2,3-linked); E = ethyl esterified N-acetylneuraminic acid (α2,6-linked). Calculation of the extraction window per analyte for MALDI-FTICR-MS data according to the formula 0.00003 ∗ "*m*/*z*" - 0.0269. This formula was obtained from the linear trendline derived from manual peak width (Full Width at Half Maximum). This list was used to extract peak areas, associated with parameters for glycan relative quantification and data quality assessment by MassyTools from the recorded spectra. All glycans not included in the final analysis are shaded gray.

Table S2. Glycosylation traits calculation formula, related to STAR Methods Calculation of glycosylation traits by using RStudio software with the same script as described in [S2] and [S3]. H = hexose; N = N-acetylhexosamine; F = deoxyhexose (fucose); Fa = Antenna fucose; S = N-acetylneuraminic acid (Sialic acid); L = lactonized N-acetylneuraminic acid (α2,3-linked); E = ethyl esterified N-acetylneuraminic acid (α2,6-linked); M = Mannose; Hy = Hybrid; A = Antenna; T = Total; C = Complex; U = Unclassifiable.

Table S3. Statistical test results, related to Figures 3, S3 and S4 and Table 2 Results of all the statistical tests performed in this manuscript. For the 3-group Kruskal-Wallis tests (Endemic Control/Asymptomatic/Visceral Leishmaniasis) as well as for each of the Wilcoxon rank-sum test comparisons a table is included. Traits meeting the requirements (significant for the Kruskal-Wallis test, significant and sufficient fold-change for the Wilcoxon test) are highlighted. The study-wide significance level was set at α = 5.0e-5 (default α of 0.05 and Bonferroni corrected by the study-wide number of tests performed, rounded up). ns = not significant, ∗p < 5.0e-5, ∗∗p < 1.0e-5, ∗∗∗p < 1.0e-6, ∗∗∗∗p < 1.0e-7. The Wilcoxon tests including the Non-endemic Controls (NC vs. EC and NC vs. ASYMP) were not included in the determination of the study-wide number of tests. As explained in the main text, the differences observed in these comparisons are not VL related and are therefore disregarded in the remainder of the study. A wider explanation can also be found below the tables.

Table S4. Receiver Operating Characteristics analysis, related to Figures 4 and S5 Receiver Operating Characteristics (ROC) analysis was performed in a group-by-group comparison for the comparisons NC-ASYMP, EC-ASYMP, EC-VL and ASYMP-VL on the discovery set and for the severity groups Uncomplicated (U-VL) - Complicated (C-VL) on the total set, because the validation set would only contain two complicated cases. Feature selection was achieved using the SES algorithm of the MXM R-package [S1] applied to Log10-normalized and scaled data on the significant and validated glycans and traits from the Wilcoxon tests. This algorithm could in theory output multiple sets of covariates for a single comparison. For each of these sets of covariates a general logistic model was calculated and the one with the lowest p-value for a Chi-square test was used in a 10-fold cross validation procedure on the discovery set. In each iteration of the cross-validation cycle the SES algorithm was applied again with the same selection procedure for covariates. From this cross-validation procedure, the Area Under the Curve (AUC) was calculated. As an independent validation the resulting models were applied to the validation set and a ROC curve was created.

Table S5. Final curated and normalized data, extracted from the MassyTools output, related to STAR Methods Data were extracted from the spectra by MassyTools. Curation scripts, as described in the text, were applied and the resulting data were normalized to the sum.
