# Supplementary material for: Total serum N-glycans mark visceral leishmaniasis in human infections with Leishmania infantum
Source: iScience. 2023 Jun 5;26(7):107021. doi: 10.1016/j.isci.2023.107021 (PMC10362369; doi:10.1016/j.isci.2023.107021)
Supplement: Document S1. Figures S1–S8 [file mmc1.pdf]

iScience, Volume *26*

Supplemental information

Total serum *N*-glycans mark visceral leishmaniasis in human infections with *Leishmania infantum*

Gabriane Nascimento Porcino, Marco René Bladergroen, Viktoria Dotz, Simone Nicolardi, Elham Memarian, Luiz Gustavo Gardinassi, Carlos Henrique Nery Costa, Roque Pacheco de Almeida, Isabel Kinney Ferreira de Miranda Santos, and Manfred Wuhrer

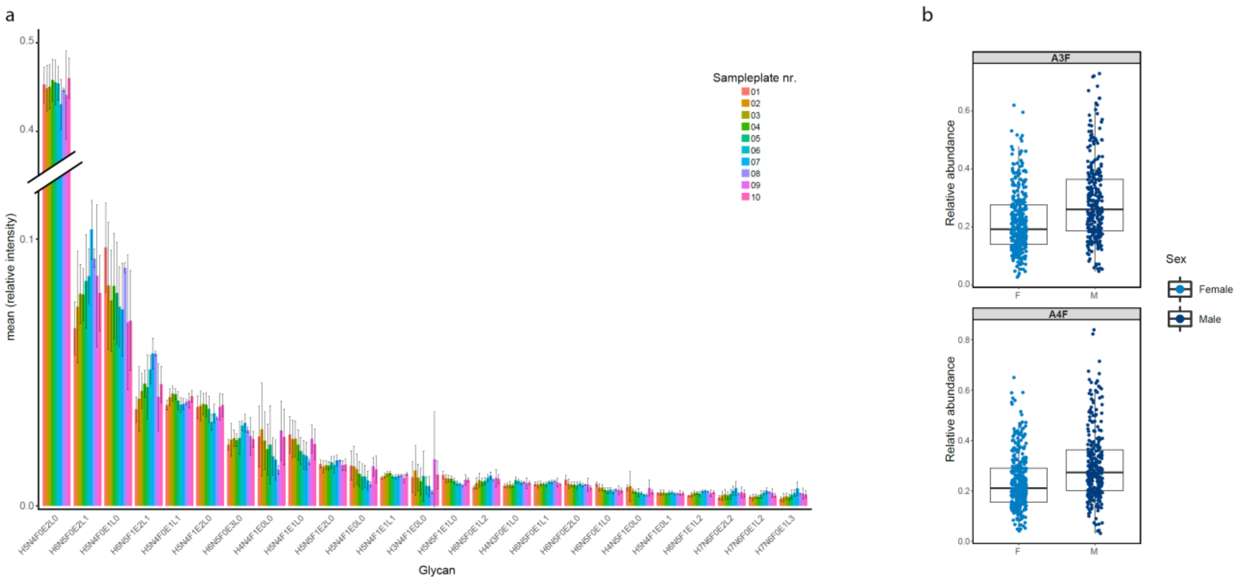

Figure S1: Repeatability and data quality of the measurements, related to STAR Methods. A) Repeatability of the automated sample preparation method and MALDI-FTICR-MS measurement for the 25 most abundant glycans as measured in the control samples (pool of serum and VisuCon). The control samples were randomly distributed over ten sample plates. The mean and standard deviation were calculated using an R-script in Rstudio software. H=hexose, N=N-acetylhexosamine, F=deoxyhexose (fucose), L=lactonized N-acetylneuraminic acid (α2,3-linked), E=ethyl esterified N-acetylneuraminic acid (α 2,6-linked). B) Sex distribution of two glycosylation traits generally known to be higher in males compared to females. The glycosylation traits show the expected behaviour, indicating that the results are not influenced by the sex disbalance in the samples

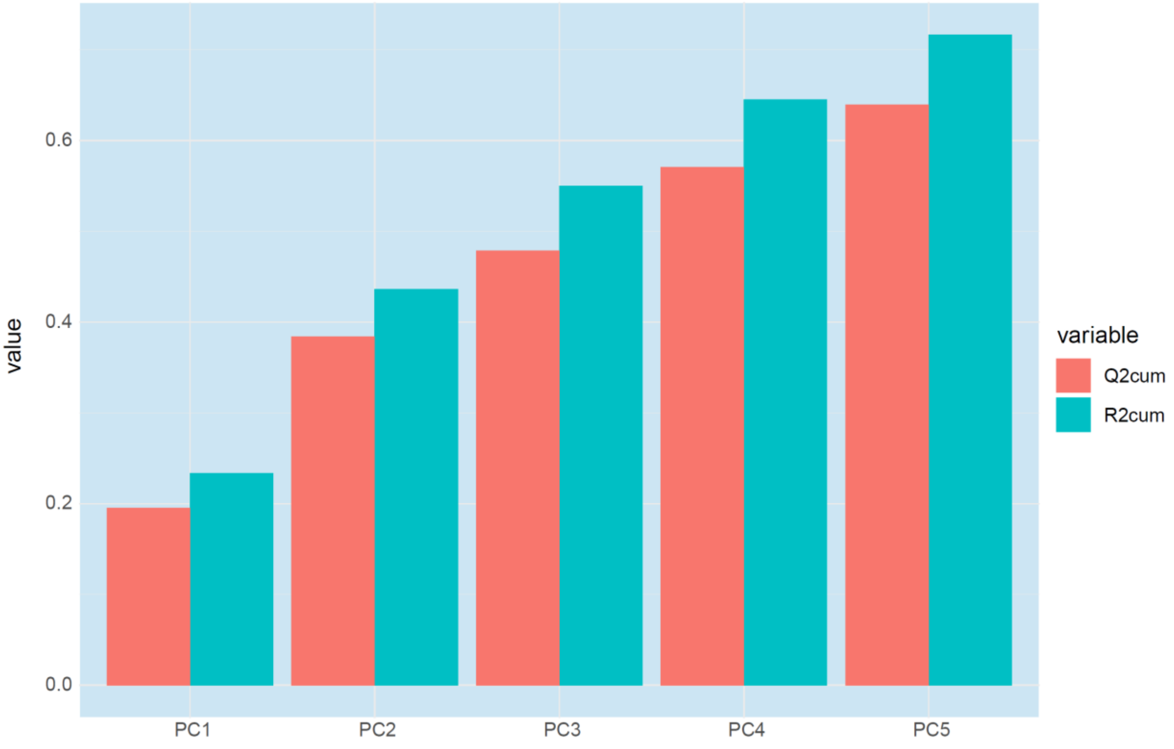

Figure S2: Summary of fit of the Principal Component Analysis (PCA). The summary is shown for the first five principal components. The fit was performed on a total of 661 serum samples with 226 variables (glycans and glycosylation traits). The variables R2cum and Q2cum represent the cumulative explained variance (goodness of fit) and a measure for the quality of the model (goodness of prediction) respectively. Statistical analysis was performed using PCA analysis with RStudio software.

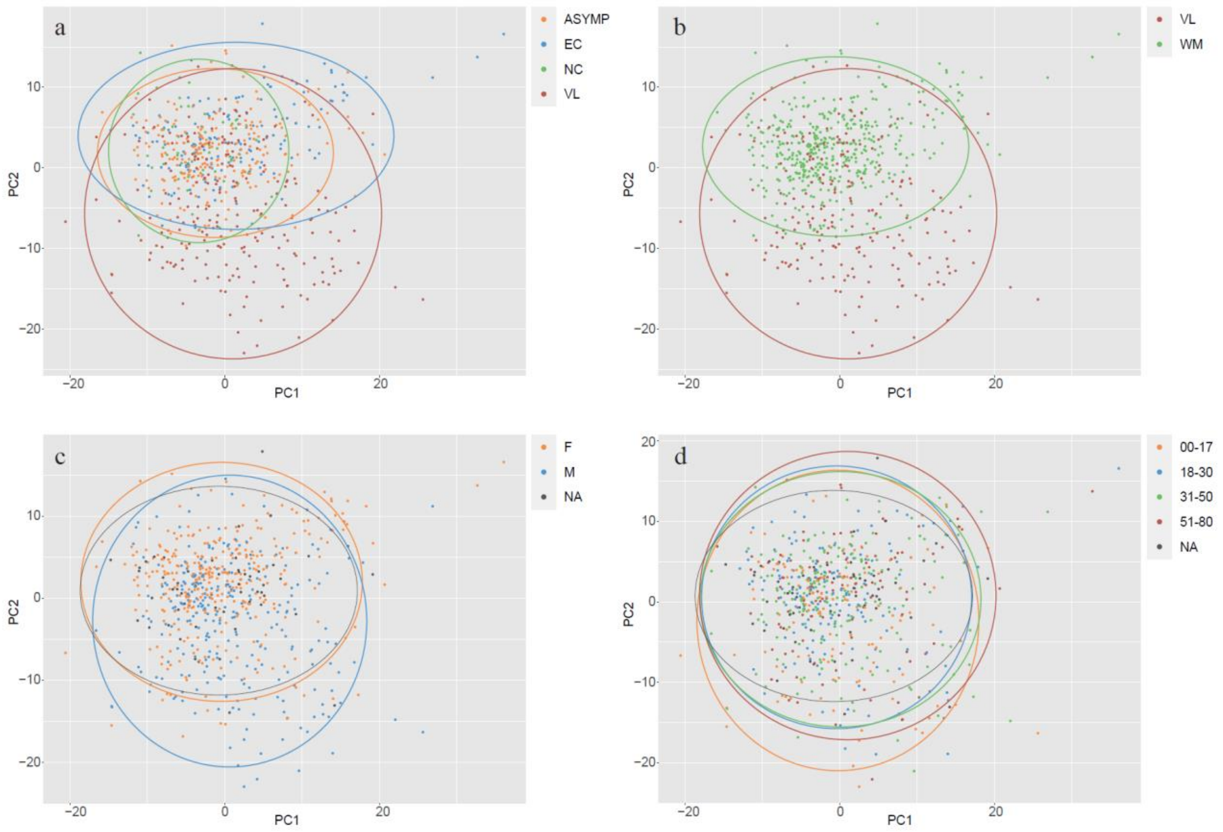

Figure S3: Score plot of the Principal Components Analysis, showing PCA 1 and PCA 2 for a model based on glycans and glycosylation traits combined. To visualize, plots were colored according to a) the four groups of the cohort, b) VL versus WM (ASYMP, NC and EC), c) sex distribution, and d) age groups. Some discrimination can be observed between VL and the other groups as well as in gender. One has to note that VL is overrepresented by males and that the gender distribution and concurrent separation in the PCA model, therefore, seems to be driven by the disease and not by gender itself. The ellipses represent the 95% confidence intervals. NC=control from non-endemic area, EC=control from endemic area, ASYMP=asymptomatic, WM=groups without manifestation of the disease, VL=Visceral Leishmaniasis, F=Female, M=Male, 00-17=Age group 0-17 years, 18-30=Age group 18-30 years, 31-50= Age group 31-50 years, 51-80=Age group 51-80 years, NA=unknown gender or age.

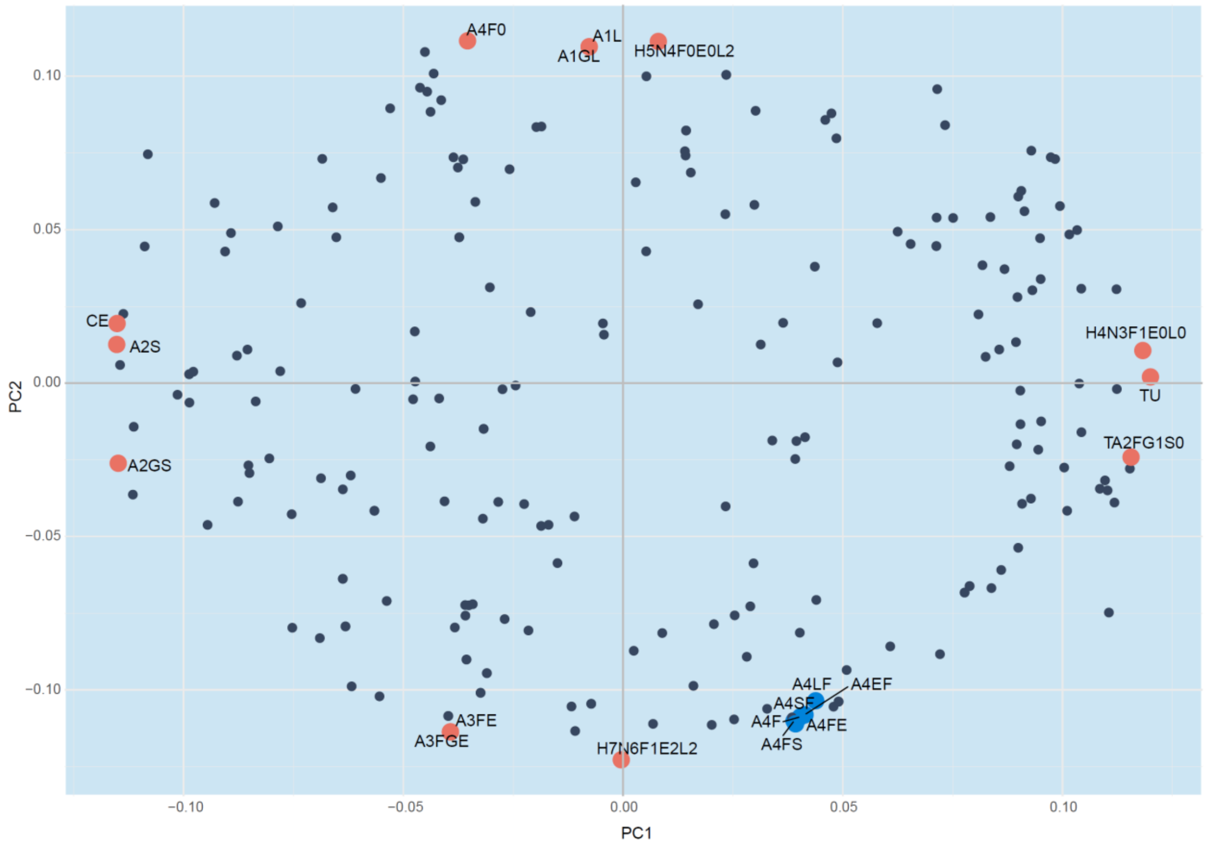

Figure S4: Loadings plot, derived from the PCA. In orange, outermost traits in either PC direction are shown, assuming these as having a large influence on the model. In light blue, traits with most influence on the model as calculated using the modeling power. Comparing with Figures 1a and 1b, the VL group is, according to the modeling power, characterized by a higher presence of tetra-antennary fucosylated glycans, while the group without any manifestation of VL (WM) is characterized by a higher presence of sialylated, non-fucosylated mono- and bi-antennary glycans. H=hexose, N=N-acetylhexosamine, F=deoxyhexose (fucose), L=lactonized N-acetylneuraminic acid (α2,3-linked), E=ethyl esterified N-acetylneuraminic acid (α2,6-linked), G=galactose, S=sialic acid, A=antenna, C=complex, T=total. Statistical analysis was performed using PCA analysis with Rstudio software.

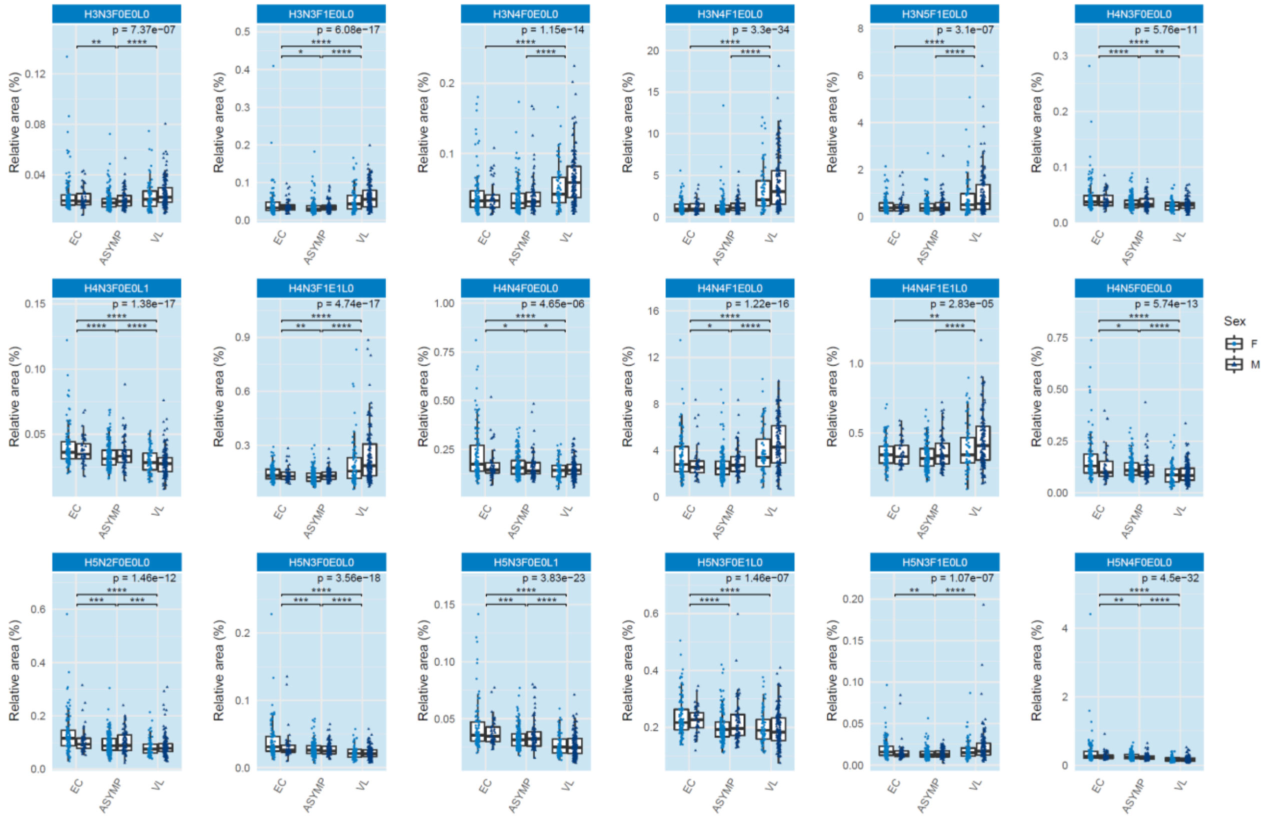

Figure S5: Changes in the N-glycome signature in active Visceral Leishmaniasis for all significant glycans and glycosylation traits. Each individual in the plots is represented by a dark blue triangle for males and by a light blue circle for females. The analysis was performed on a total of 73 glycans and 153 glycosylation traits using a Kruskal-Wallis test and post-hoc Dunn's test with a significance threshold of α=5.0e-5. EC=Endemic Control, ASYMP=Asymptomatic and VL=Visceral Leishmaniasis. H=hexose, N=N-acetylhexosamine, F=deoxyhexose (fucose), L=lactonized N-acetylneuraminic acid (α2,3-linked), E=ethyl esterified N-acetylneuraminic acid (α2,6-linked), A=antenna, T=total. *p<5.0e-5, **p<1.0e-5, ***p<1.0e-6, ****p<1.0e-7.

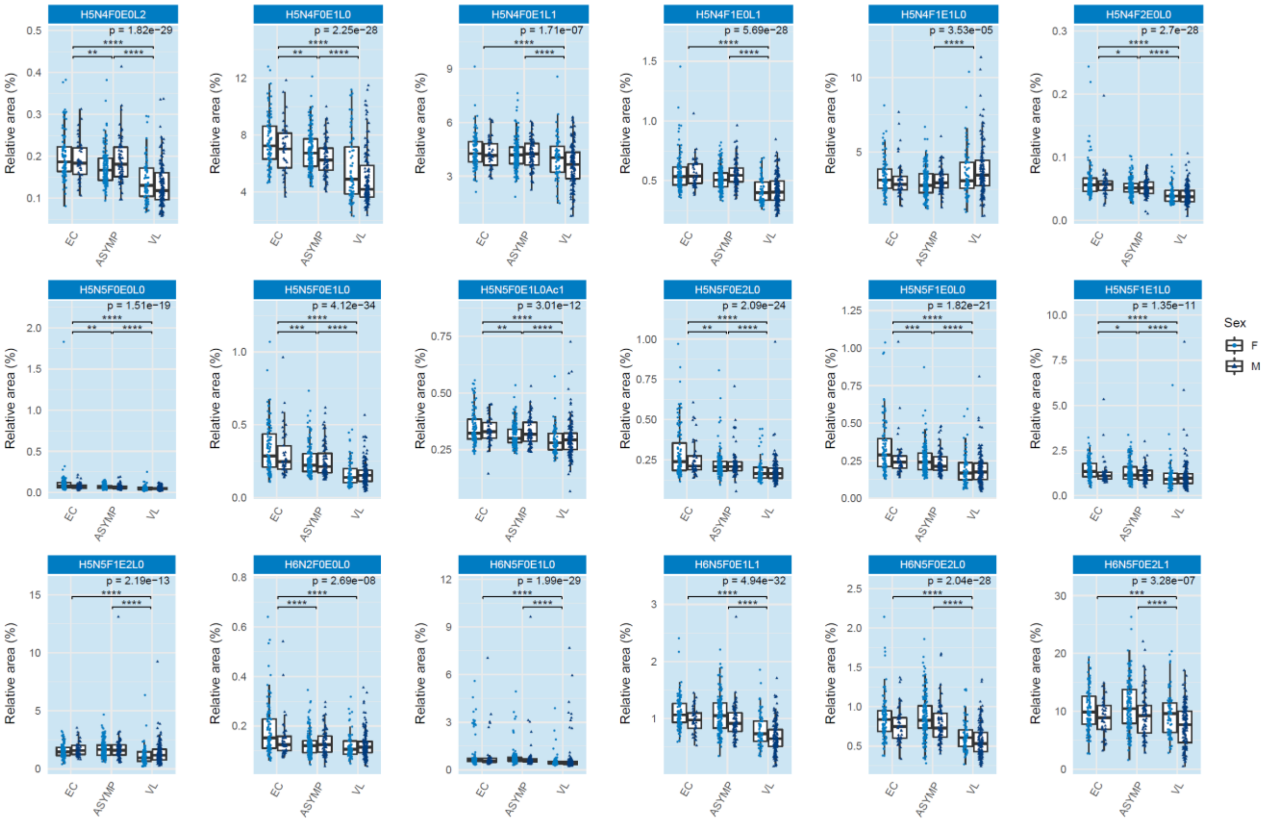

Figure S5 (continued)

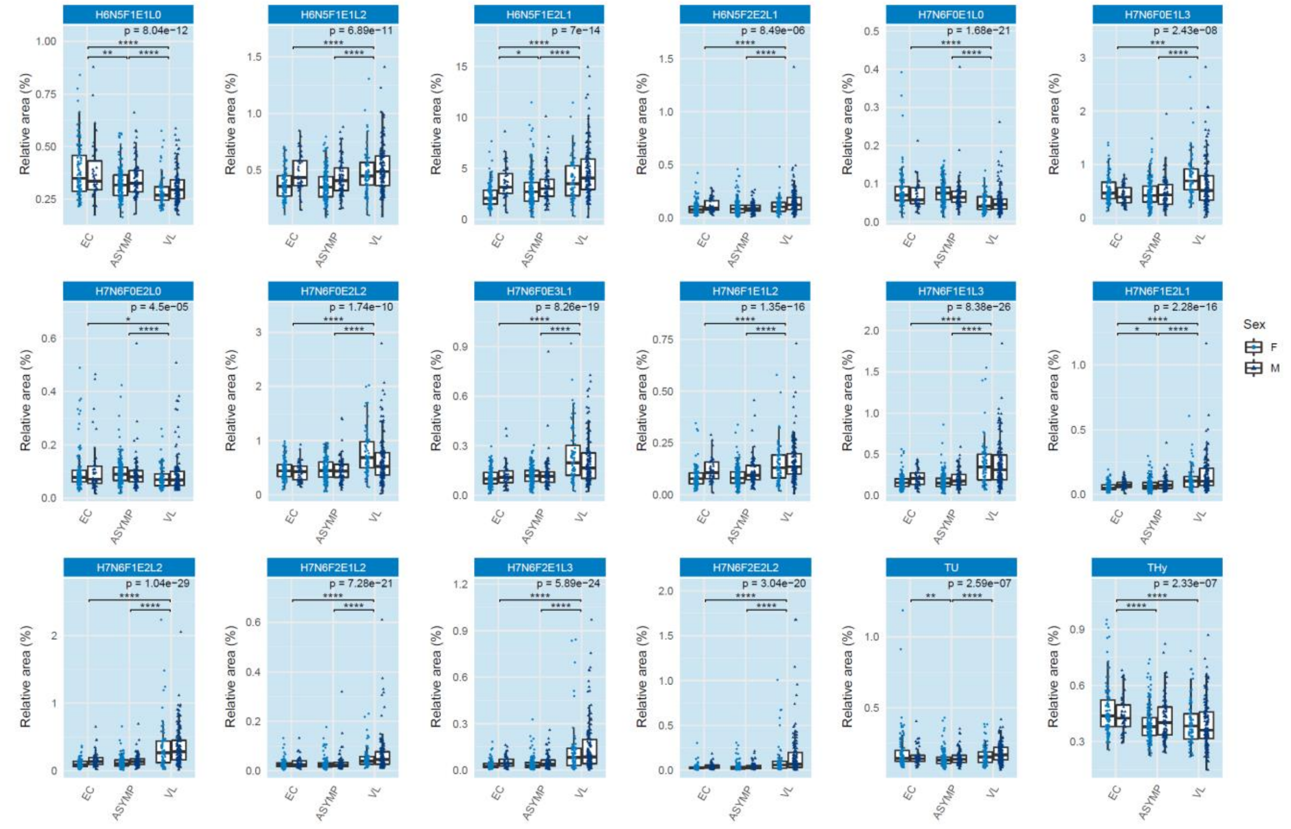

Figure S5 (continued)

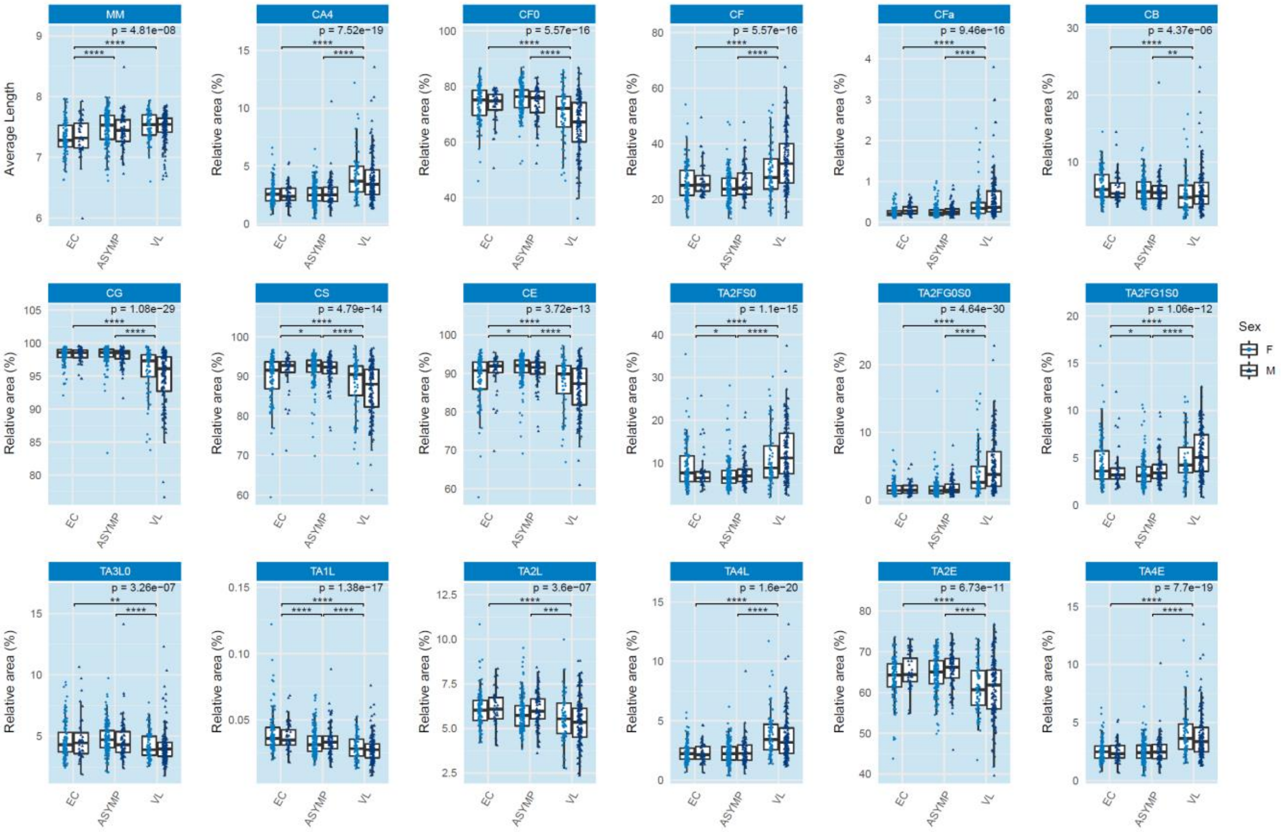

Figure S5 (continued)

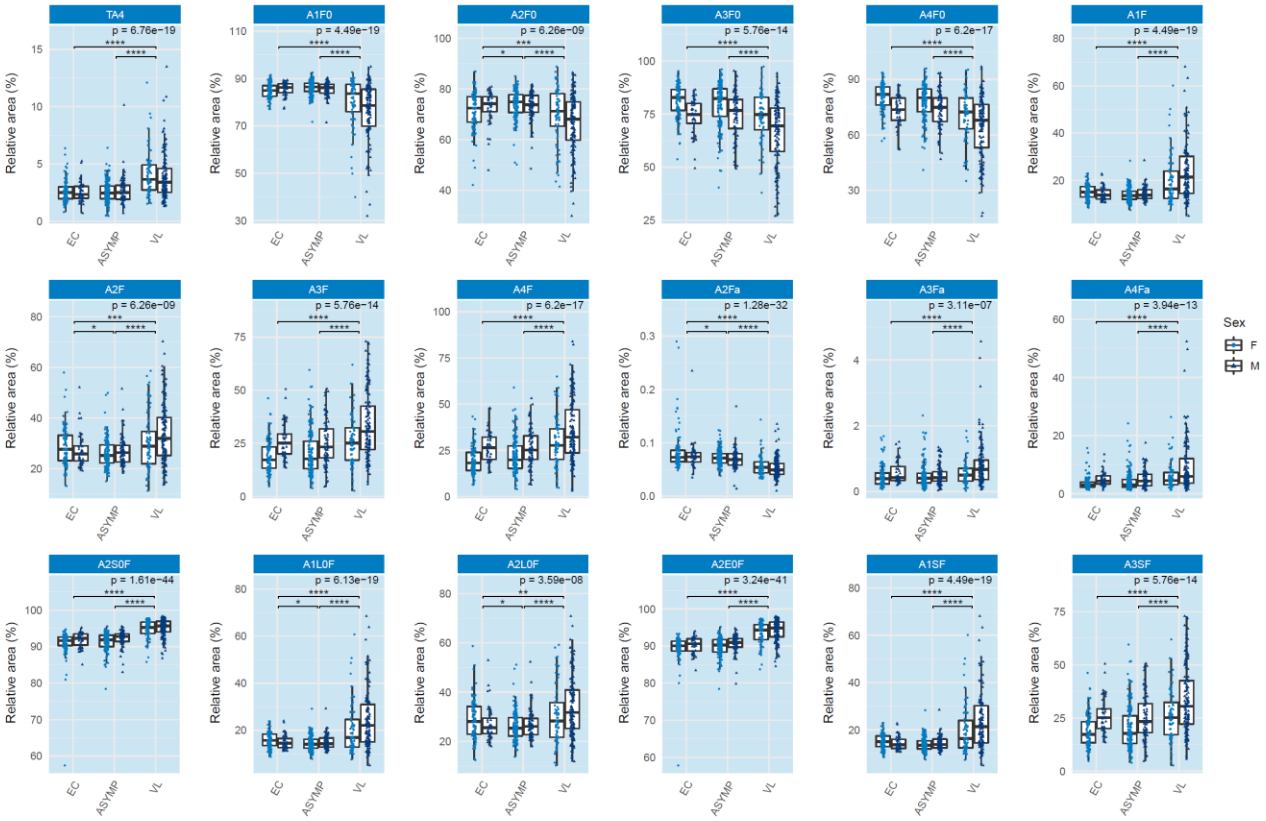

TA4
A1F0
A2F0
A3F0
A4F0
A1F
A2F
A3F
A4F
A2Fa
A3Fa
A4Fa
A2S0F
A1L0F
A2L0F
A2E0F
A1SF
A3SF
Relative area (%)
EC
ASYMP
VL
Sex
F
M

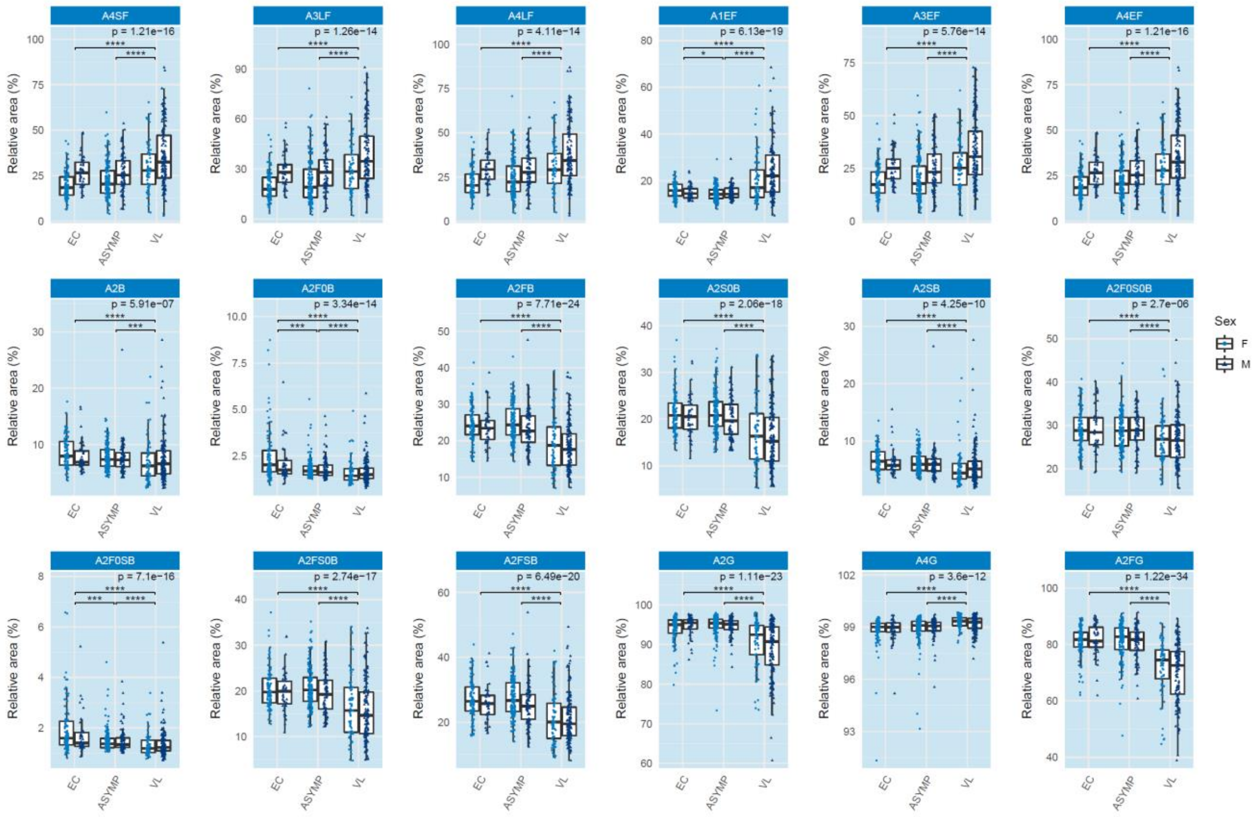
A4SF
p = 1.21e−16
A3LF
p = 1.26e−14
A4LF
p = 4.11e−14
A1EF
p = 6.13e−19
A3EF
p = 5.76e−14
A4EF
p = 1.21e−16
A2B
p = 5.91e−07
A2F0B
p = 3.34e−14
A2FB
p = 7.71e−24
A2S0B
p = 2.06e−18
A2SB
p = 4.25e−10
A2F0S0B
p = 2.7e−06
A2F0SB
p = 7.1e−16
A2FS0B
p = 2.74e−17
A2FSB
p = 6.49e−20
A2G
p = 1.11e−23
A4G
p = 3.6e−12
A2FG
p = 1.22e−34
Relative area (%)
EC
ASYMP
VL
Sex
F
M

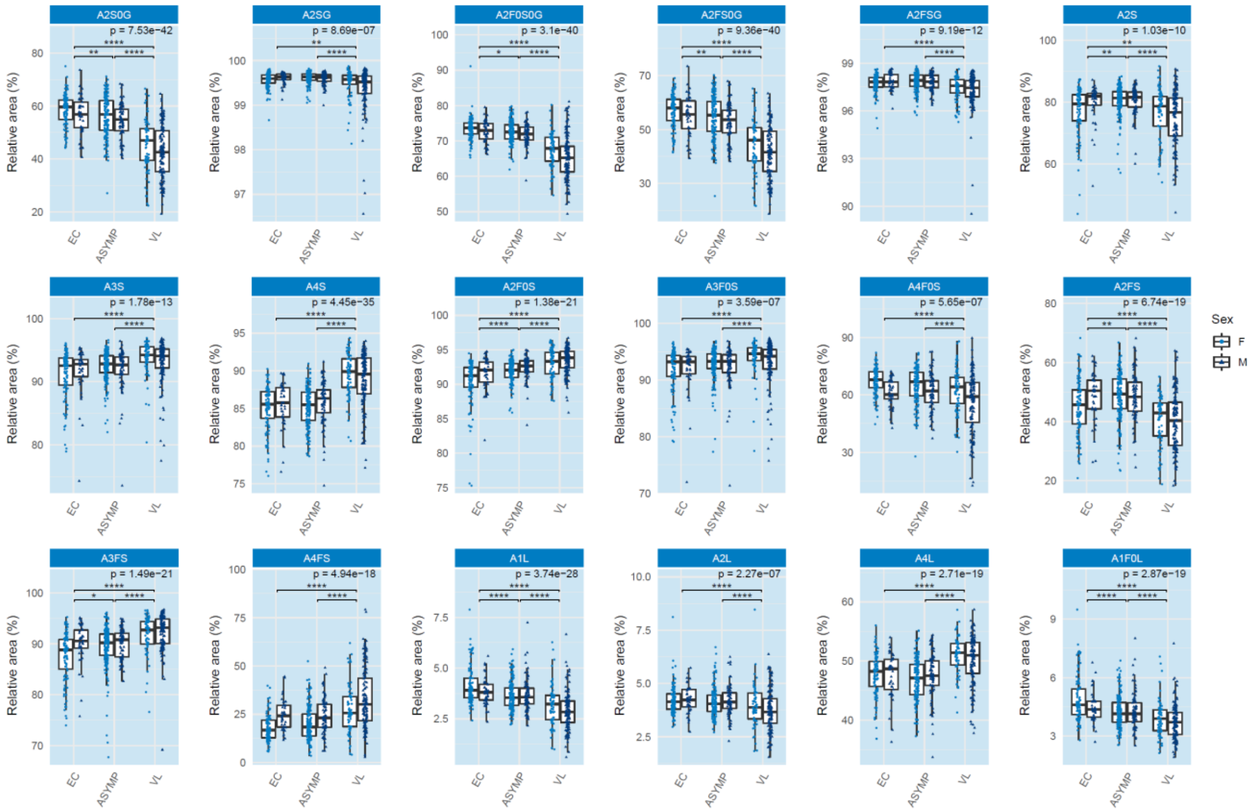

Figure S5 (continued)

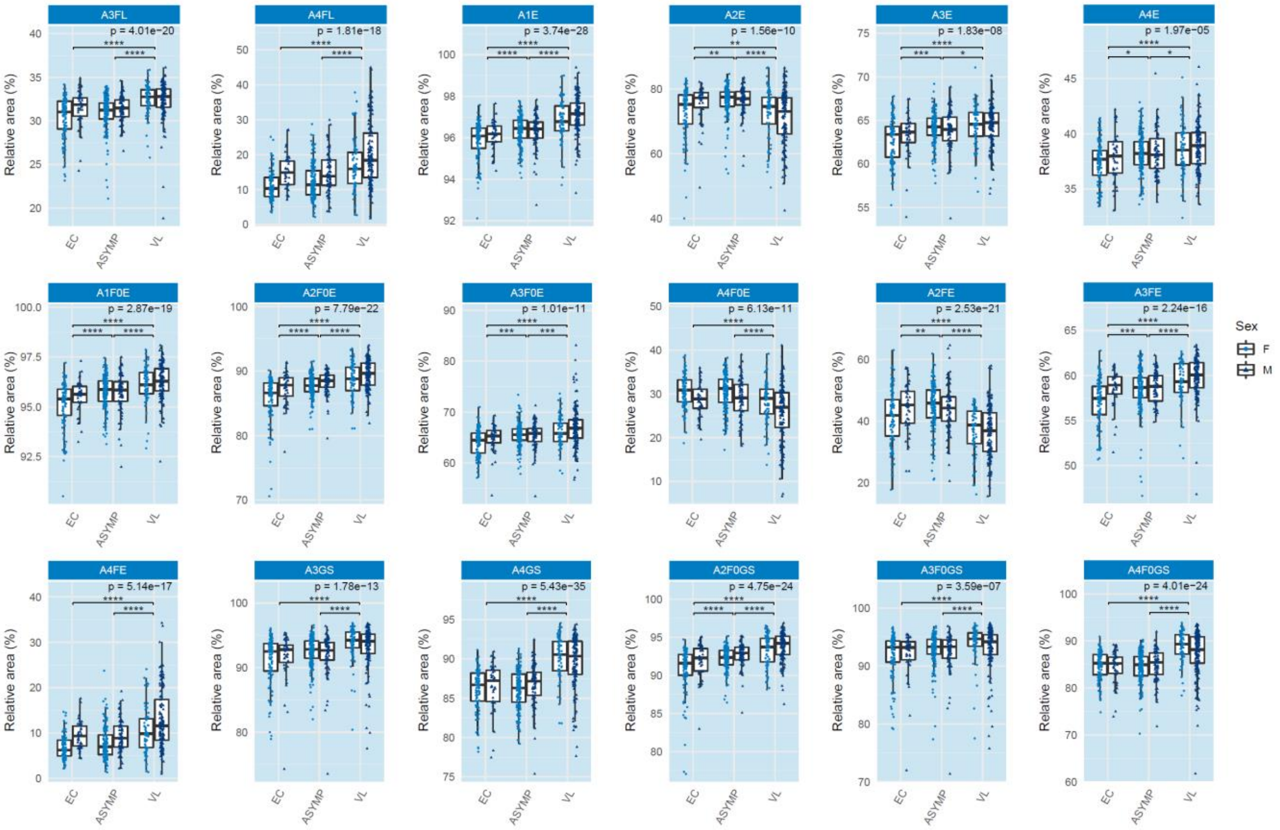
A3FL
p = 4.01e−20
A4FL
p = 1.81e−18
A1E
p = 3.74e−28
A2E
p = 1.56e−10
A3E
p = 1.83e−08
A4E
p = 1.97e−05
A1F0E
p = 2.87e−19
A2F0E
p = 7.79e−22
A3F0E
p = 1.01e−11
A4F0E
p = 6.13e−11
A2FE
p = 2.53e−21
A3FE
p = 2.24e−16
A4FE
p = 5.14e−17
A3GS
p = 1.78e−13
A4GS
p = 5.43e−35
A2F0GS
p = 4.75e−24
A3F0GS
p = 3.59e−07
A4F0GS
p = 4.01e−24
Relative area (%)
EC
ASYMP
VL
Sex
F
M

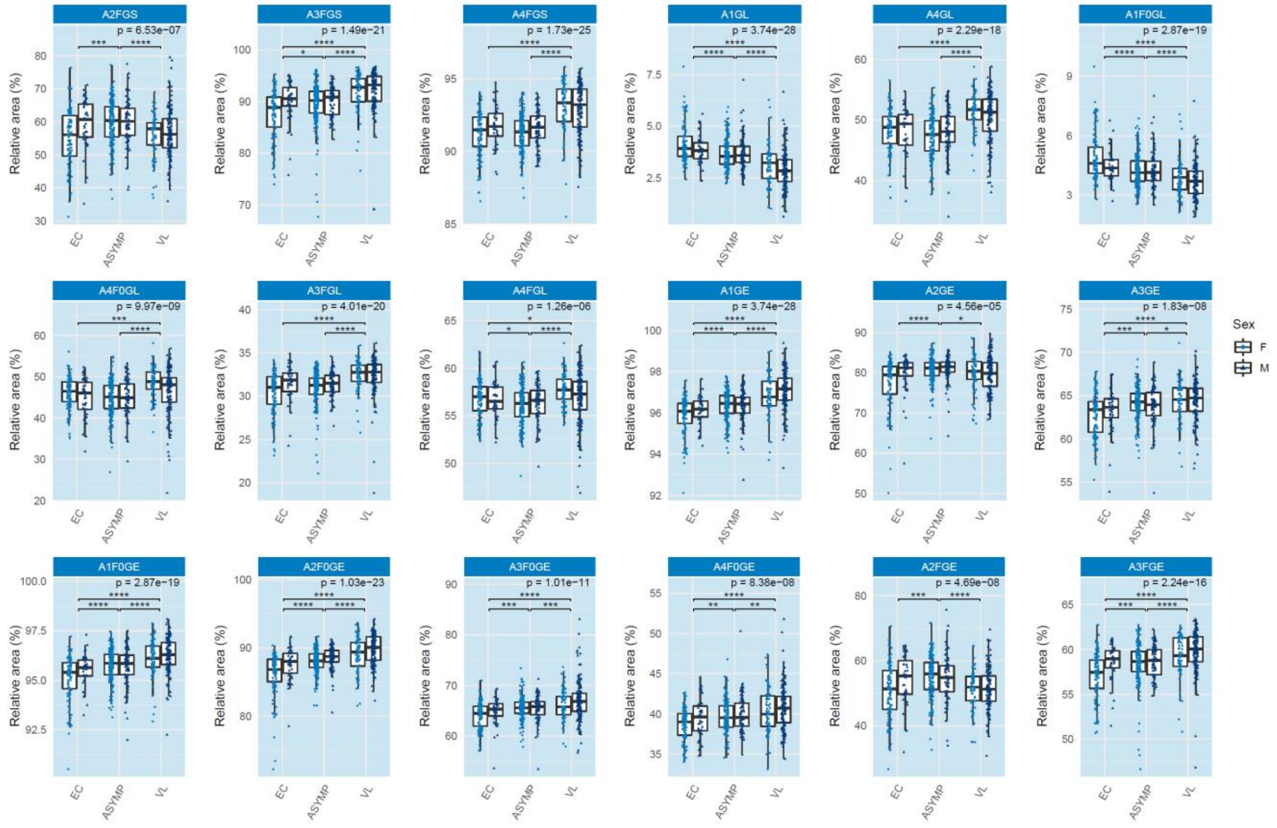

Figure S5 (continued)

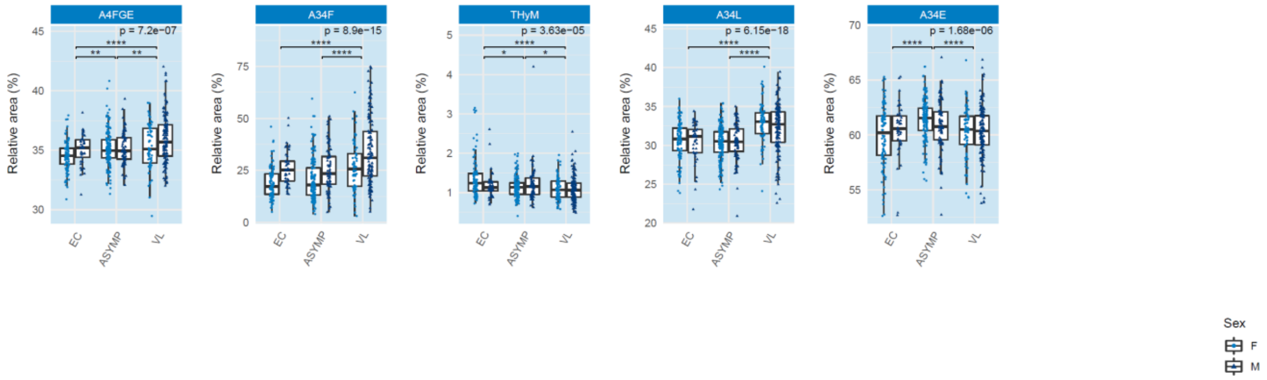

Figure S5 (continued)

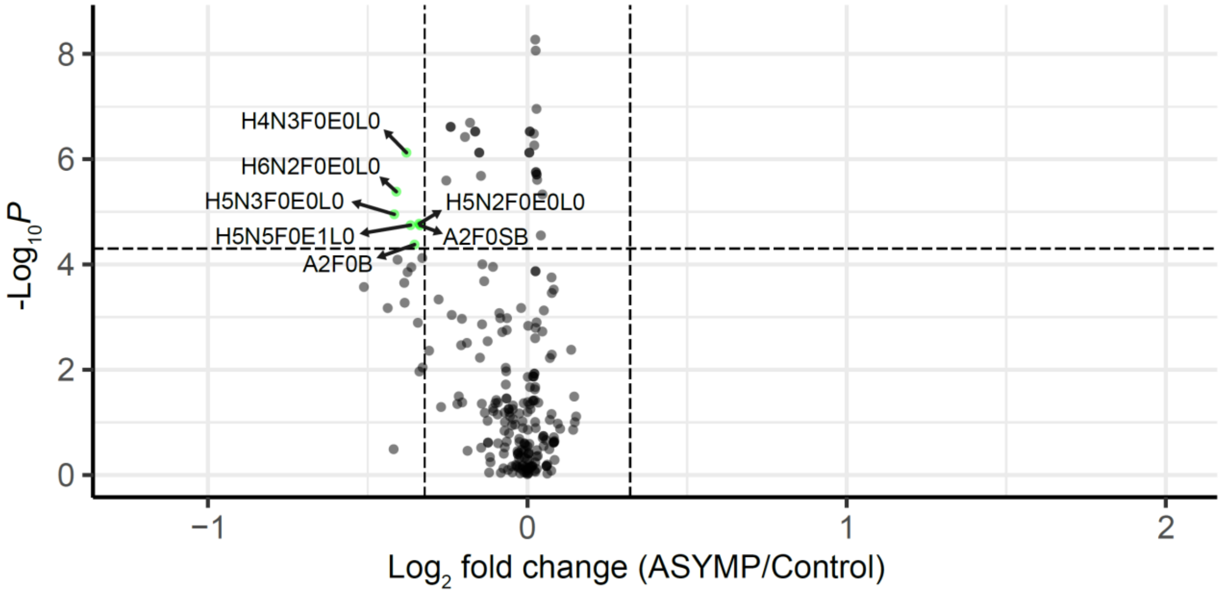

Figure S6: Differential glycan expression between control and asymptomatic individuals (ASYMP). Values on the x- and y-axes show Log2(fold change) vs. -Log10(p-value) of a Wilcoxon rank sum test respectively. Green dots indicate significant decrease (α=5.0e-5) with at least an absolute log2(fold-change) of 0.3, while grey dots indicate no significant or sufficient change. The analysis was performed on a total of 226 variables (glycans and glycosylation traits). The complete table of the Wilcoxon tests can be found in Supplementary Table S4. H=hexose, N=N-acetylhexosamine, F=deoxyhexose (fucose), L=lactonized N-acetylneuraminic acid (α2,3-linked), E=ethyl esterified N acetylneuraminic acid (α2,6-linked), A=antenna, B=bisected, S=sialic acid.

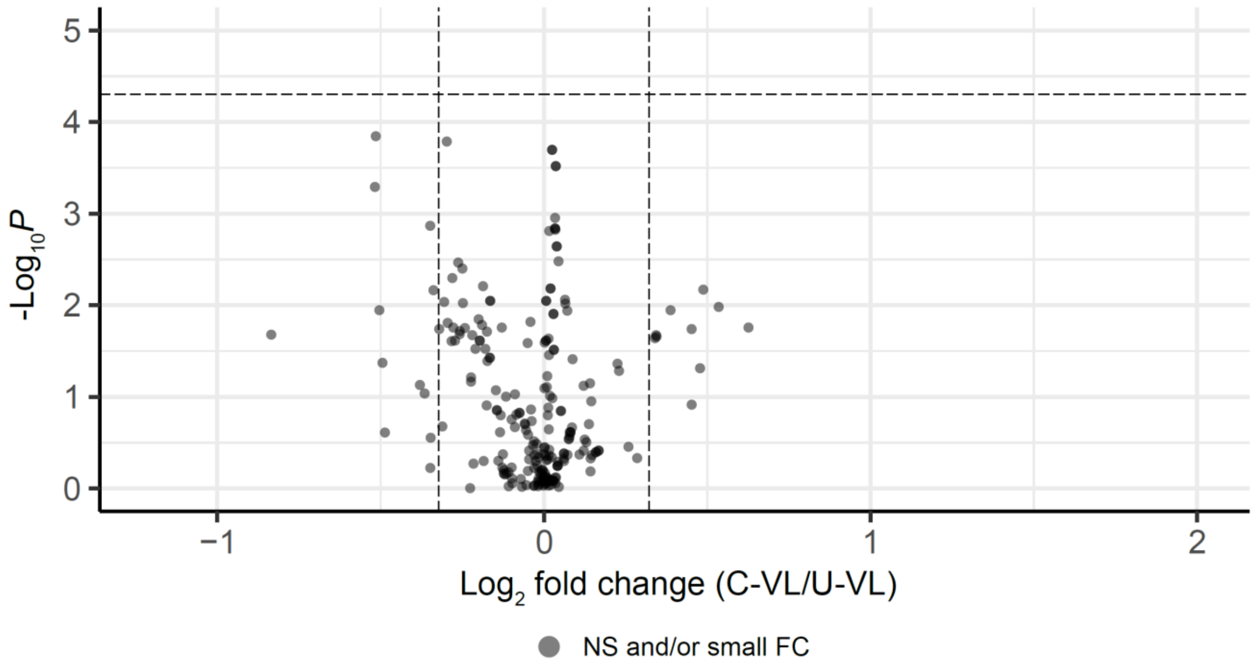

Figure S7: Differential glycan expression between uncomplicated and complicated VL. Values on the x- and y-axes show Log2(fold change) vs. -Log10(p-value) of a Wilcoxon rank sum test respectively. The alpha for the Wilcoxon test was set at 5.0e-5, resulting in no significant differences between patients with discriminating disease severity.

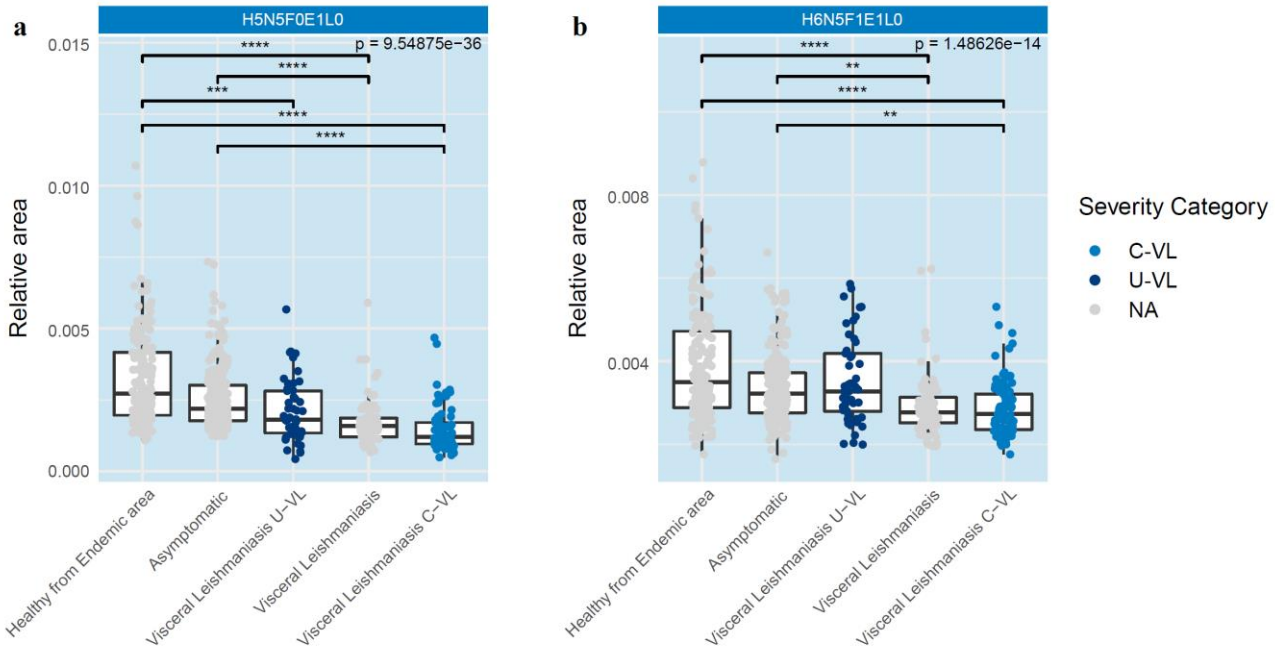

Figure S8: Glycan differences with clinical severity. In this figure two glycans (H5N5F0E1L0 (a) and H6N5F1E1L0 (b)) which are possibly influenced by disease severity as described in the text, are more extensively compared between the subgroups of investigation. Although not statistically different between the two severity groups (uncomplicated (U-VL, dark blue circle) and complicated (C-VL, light blue circle)), these glycans were shown to be statistically different in VL compared to controls from endemic area and asymptomatic patients (gray circles). For H5N5F0E1L0 a gradual reduction could be observed with increasing disease severity (with the overall VL group as ‘intermediate’ or ‘mean’ between U-VL and C-VL). H6N5F1E1L0 seems to be reduced slightly (but not significantly) upon infection but reduces significantly when VL becomes complicated. The analysis was performed using a Kruskal-Wallis test and post-hoc Dunn’s test with a significance threshold of α=5.0e-5. H=hexose, N=N-acetylhexosamine, F=deoxyhexose (fucose), L=lactonized N-acetylneuraminic acid (α2,3-linked), E=ethyl esterified N-acetylneuraminic acid (α2,6-linked). *p<5.0e-5, **p<1.0e-5, ***p<1.0e-6, ****p<1.0e-7.
